# Tumor Suppressor WWOX Contributes to the Elimination of Tumorigenic Cells in *Drosophila melanogaster*

**DOI:** 10.1371/journal.pone.0136356

**Published:** 2015-08-24

**Authors:** Louise V. O’Keefe, Cheng Shoou Lee, Amanda Choo, Robert I. Richards

**Affiliations:** Department of Genetics and Evolution, Centre for Molecular Pathology, School of Biological Sciences, The University of Adelaide, Adelaide, Australia; University of Dayton, UNITED STATES

## Abstract

*WWOX* is a >1Mb gene spanning *FRA16D* Common Chromosomal Fragile Site, a region of DNA instability in cancer. Consequently, altered *WWOX* levels have been observed in a wide variety of cancers. *In vitro* studies have identified a large number and variety of potential roles for WWOX. Although its normal role *in vivo* and functional contribution to cancer have not been fully defined, WWOX does have an integral role in metabolism and can suppress tumor growth. Using *Drosophila melanogaster* as an *in vivo* model system, we find that WWOX is a modulator of TNFα/Egr-mediated cell death. We found that altered levels of WWOX can modify phenotypes generated by low level ectopic expression of TNFα/Egr and this corresponds to altered levels of Caspase 3 activity. These results demonstrate an *in vivo* role for WWOX in promoting cell death. This form of cell death is accompanied by an increase in levels of reactive oxygen species, the regulation of which we have previously shown can also be modified by altered WWOX activity. We now hypothesise that, through regulation of reactive oxygen species, WWOX constitutes a link between alterations in cellular metabolism observed in cancer cells and their ability to evade normal cell death pathways. We have further shown that WWOX activity is required for the efficient removal of tumorigenic cells from a developing epithelial tissue. Together these results provide a molecular basis for the tumor suppressor functions of WWOX and the better prognosis observed in cancer patients with higher levels of WWOX activity. Understanding the conserved cellular pathways to which WWOX contributes provides novel possibilities for the development of therapeutic approaches to restore WWOX function in cancer.

## Introduction

Evasion of cell death and altered metabolism are recognized as hallmarks of cancer cells, whilst DNA instability is one of the enabling characteristics [1]. The *FRA16D* Common Chromosomal Fragile Site (CCFS) spanning gene, *WW domain containing oxidoreductase (WWOX)*, participates in each of these phenomena and therefore its perturbation in cancer cells presents multiple possible avenues for contributing to cancer cell biology. CCFS are specific regions of chromosomes that can be induced to break *in vitro* by inhibitors of DNA polymerase and are affected by certain dietary or environmental factors [2–3]. More than 70 common fragile sites have been identified in the human genome and it has been observed that there is a hierarchy of sensitivity *in vitro* that is matched by the frequency with which these sites show *in vivo* DNA instability in various cancers [4]. Of these fragile sites, *FRA3B* and *FRA16D* have been shown to be the most frequent regions of recurrent homozygous deletion in cancer cell lines [5]. CCFS are typically located within extremely large genes (i.e. *FRA3B* in 1.5 Mb *FHIT* gene, *FRA16D* in 1.1 Mb *WWOX* gene), a relationship that is conserved in mice and suggestive of biological significance [4, 6]. DNA instability at these sites, resulting in deletion(s) and / or localised rearrangements, is associated with alterations to CCFS-associated gene expression [7–8].

Altered expression of *WWOX* has been reported for many different cancer cell types (reviewed in [9–11]). In addition, low expression alleles of *WWOX* were found at a higher frequency in patients with lung cancer [12] or glioma [13], consistent with decreased *WWOX* as a predisposing factor for tumorigenesis. *WWOX* hypomorphic mice showed an increased incidence of B-cell lymphoma [14] and mice heterozygous for *WWOX* exhibit higher rates of tumor growth [15], however the tumor cells still express WWOX protein indicating a lack of the typical ‘second-hit’ somatic mutation that is characteristic of classical tumor suppressors. Thus it appears that a reduced level of WWOX activity is sufficient for contribution to cancer progression. Conversely, ectopically expressed WWOX has been shown to function as a suppressor of tumor growth since restoration of WWOX activity in cancer cells inhibits their growth *in vivo* [16–20]. Correlation of higher WWOX expression with better prognosis has also been reported for various types of cancer including colon, breast and bladder [21–23]. Therefore the pathways that WWOX normally participates in, and the nature of this participation, are of considerable interest for their likely causal and therapeutically targetable contribution to cancer cell biology.

*WWOX* encodes an enzyme with short-chain dehydrogenase/reductase (SDR) activity in addition to two WW domains that facilitate protein-protein interactions. WWOX has been implicated in a diverse range of cellular pathways and processes in mammalian studies by virtue of its physical and / or functional interactions with other proteins or pathways (reviewed in [24–26]). Whilst various functions for WWOX have been revealed *in vitro*, it is difficult to assess their relative significance and contribution to cancer *in vivo*. A role for WWOX in metabolism has been established through the analysis of knockout models in mouse, rat and *D*. *melanogaster* [14–15,27–30]. The protein encoded by *WWOX* has been found not only to contribute to cellular metabolism but also is, in turn, regulated by the relative level of glycolysis versus oxidative phosphorylation [31]. WWOX has also been widely reported to play a role in apoptotic pathways, principally through interactions with the tumor suppressor p53 (reviewed in [24–32]). A pro-apoptotic role for WWOX *in vitro* has previously been reported for many different cancer cell types; multiple myeloma [33], colon [34], gall bladder [35], cervical [36], leukaemic [37], glioblastoma [38–39], hepatoma [40], lung [17], pancreatic [18] and squamous epithelia [41]. However the molecular mechanism(s) by which WWOX contributes to cell death pathways *in vivo* has not been determined. The genetically tractable system of *D*. *melanogaster* is an effective system in which to dissect various aspects of the contribution of WWOX to cellular pathways. Herein we determine the role of WWOX in modulating TNFα-mediated cell death through regulation of Caspase 3 activity. In addition we are able to demonstrate a requirement for WWOX in the elimination of tumorigenic cells, thus supporting a requirement for WWOX function early in the tumorigenic process for the removal of abnormal cells.

## Results

### Altered WWOX modulates ectopic Egr/TNFα eye phenotypes

**Fig 1:**
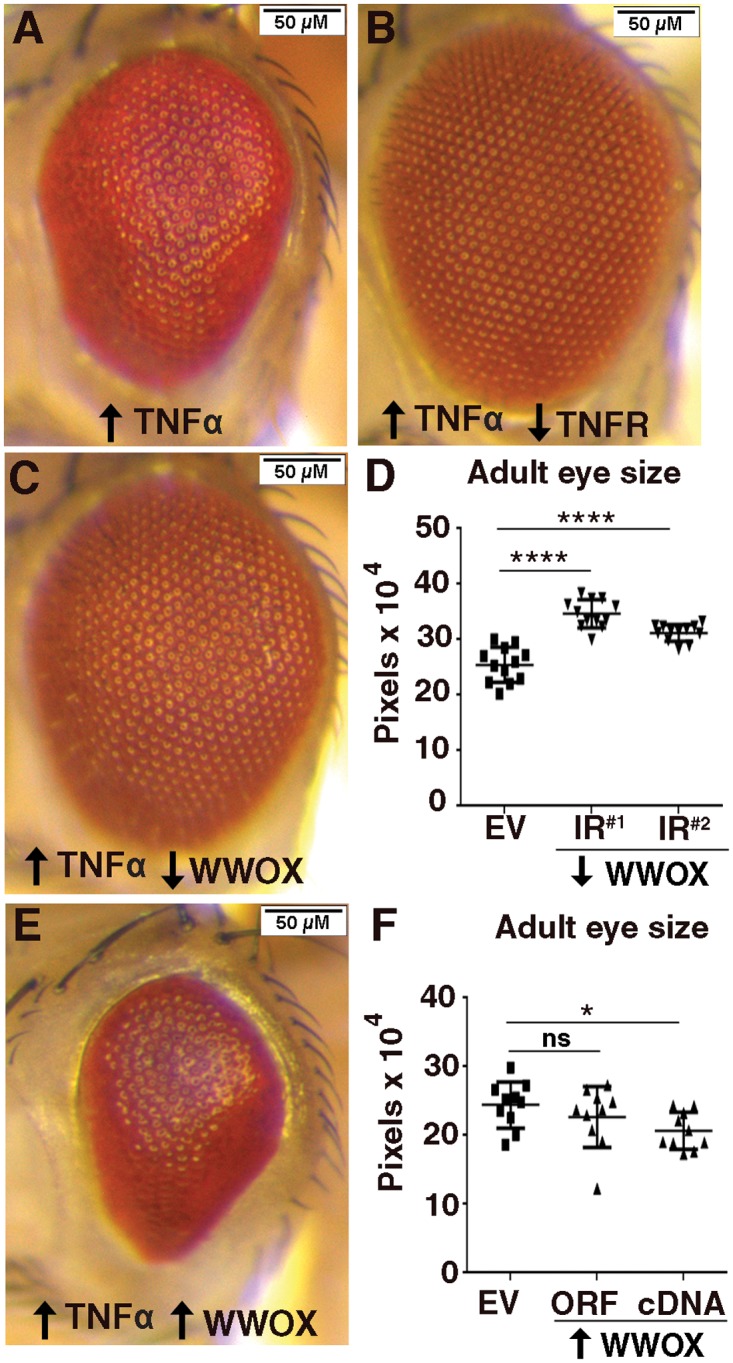
Altered WWOX modifies ectopic Egr/TNFα-mediated eye phenotype. **(A)** Ectopic expression of Egr/TNFα (GMR>*egr* ^*+w*^ *>empty vector (EV)*) results in a decrease in eye size and disruption to ommatidial patterning. **(B)** The ectopic Egr/TNFα phenotype is completely suppressed by decreased levels of TNFR (GMR>*egr* ^*+w*^ *>wengen/TNFR-IR*). **(C)** Decreased expression of WWOX by RNAi knockdown (*GMR>egr* ^*+w*^ *>WWOX-IR* ^*#1*^) results in suppression of the rough eye phenotype. **(D)** Quantification of increased eye size with independent WWOX knockdown constructs (*GMR>egr* ^*+w*^ *>WWOX-IR* ^*#1*^ and *GMR>egr* ^*+w*^ *>WWOX-IR* ^*#2*^ *)*. **(E)** Increased expression of WWOX by ectopic expression of the WWOX cDNA (GMR>*egr* ^*+w*^ *>WWOX-cDNA*) resulted in an enhancement of the Eiger/TNFα phenotype. **(F)** Quantification of decreased eye size with independent ectopic expression constructs for WWOX (GMR>*egr* ^*+w*^ *>WWOX-ORF* and GMR>*egr* ^*+w*^ *>WWOX-cDNA)*. Significance indicated by **** = p <0.0001, * = p<0.05 and ns = not significant.

**Fig 2:**
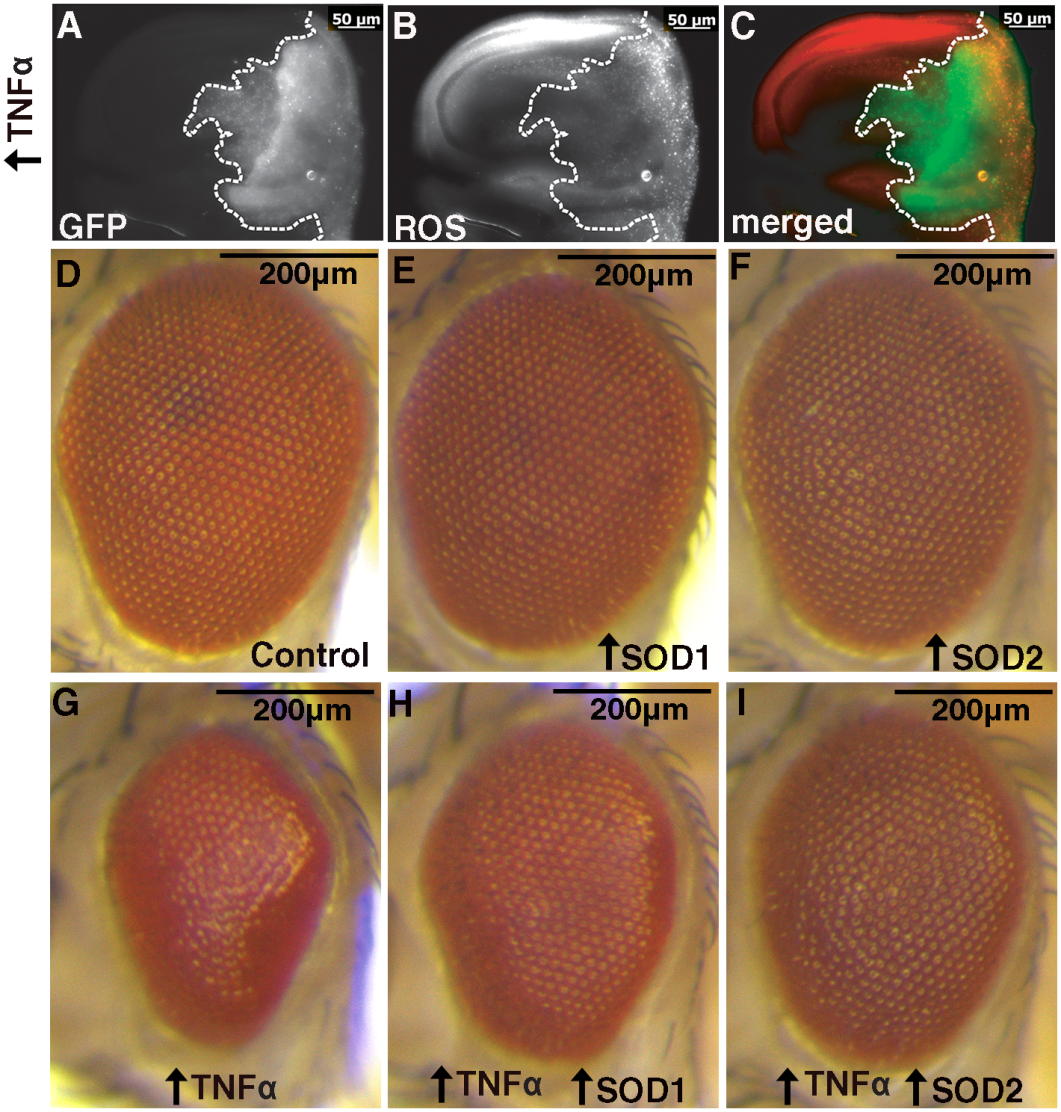
Ectopic expression of Egr/TNFα gives increased ROS and is suppressed by increased SOD activity. **(A)** Ectopic expression of GFP and Egr/TNFα with *hh-GAL4* in the posterior portion of wing discs of wandering third instar larvae, GFP shows the region of ectopic expression (outlined by dotted line). **(B)** Punctate CellRox staining revealed increased ROS levels at posterior edge of disc. **(C)** Merged image with GFP in green and CellRox in red. **(D)** Control eye phenotype (*GMR>EV*) showing regular ordered arrays of ommatidial units on the surface of the adult eye **(E-F)** Ectopic expression of SOD1 or SOD2 alone has no effect. **(G)**. Ectopic expression of Egr/TNFα in the eye (GMR>*egr* ^*+w*^ *>EV*) results in a decrease in eye size and disruption to ommatidial patterning. **(H-I)** The ectopic Egr/TNFα phenotype is obviously suppressed by increased levels of SOD1 or SOD2.

Ectopically expressed WWOX has been shown to enhance the *in vitro* cytotoxicity of TNFα in various tissue culture cell lines [42], yet the contribution of WWOX to TNFα-mediated cell death *in vivo* has not been determined. *D*. *melanogaster* has a single ortholog to TNFα encoded by the *EDA-like cell death trigger* or *Eiger (Egr)* gene [43–44]. Genetic modification analyses have previously revealed a number of metabolic genes that are rate-limiting in their contribution to Egr/TNFα-induced cell death phenotypes in the *D*. *melanogaster* eye [45]. The *WWOX* gene has been identified as participating in aerobic metabolism in *D*. *melanogaster* [30] and thus also represents a candidate for contributing to Egr/TNFα-mediated cell death. Ectopic over-expression of a low level expression construct for Egr/TNFα in the eye during its development results in a phenotype characterised by disruption to the precise patterning of repeated ommatidial units on the external surface of the eye as well as a decrease in overall size (Fig 1A and [46]) when compared to a control eye (Fig 2D). This Egr/TNFα phenotype was completely suppressed by RNAi-mediated knockdown of *wengen*, a gene that encodes the *D*. *melanogaster* TNF receptor (*TNFR*) thus confirming the specificity of the ectopic Egr/TNFα-mediated phenotype (Fig 1B and [47–48]). Introduction of a *WWOX* knockdown construct (WWOX-*IR* ^*#1*^) resulted in suppression of the Egr/TNFα-mediated rough eye phenotype evident as restoration of ommatidial patterning across the surface of the eye as well as an increase in eye size (Fig 1C and 1D). A similar suppression of eye size was observed with an independent *WWOX* knockdown construct as well as in flies heterozygous for *WWOX* loss-of-function mutant alleles (S1 Fig). This indicates that WWOX can contribute to low level Egr/TNFα-mediated cell death.

Ectopic expression of WWOX alone does not result in any obvious cell death-induced phenotype in the biological context of the *D*. *melanogaster* eye (S1 Fig). Ectopic over-expression of WWOX cDNA showed enhancement of the Egr/TNFα mediated mild rough eye phenotype evident as further disruption to ommatidial patterning and a significant decrease in eye size (Fig 1E and 1F). A decrease in adult eye size was also observed with ectopic over-expression of an open reading frame (ORF) encoding WWOX although these results were more variable despite comparable levels of expression of WWOX (Fig 1F and S1 Fig). Notably, ectopic expression of WWOX together with Egr/TNFα did not result in any further increase in WWOX levels (S1 Fig). Together these data demonstrate that WWOX contributes to Egr/TNFα-mediated cell death phenotype.

To determine whether the interaction between WWOX and Egr/TNFα was specific we also tested the contribution of WWOX with other inducers of cell death. Given the significant analysis of WWOX function together with p53 in the literature, we also tested for any modification of ectopic p53 phenotypes with altered levels of WWOX. However, we were unable to detect any alteration to the much more severe eye phenotypes generated by ectopic expression of either p53 or Hid (head involution defective), another of the cell death promoting proteins identified in *D*. *melanogaster* (S2 Fig).

### Egr/TNFα-mediated cell death phenotypes are mediated by increased ROS

Reactive oxygen species (ROS) are known to be principle effector molecules of Egr/TNFα-mediated cell death [45]. We were able to confirm this in larval wing discs expressing Egr/TNFα in the posterior region by increased staining for CellRox compared to low levels observed in the anterior control region for each disc (Fig 2A–2C). We also determined whether the Egr/TNFα-mediated rough eye phenotypes can be modified by enzymes known to modify ROS levels. Superoxide dismutase (SOD) activity is required for conversion of superoxide to hydrogen peroxide as an intermediary in the detoxification process. There are different SOD enzymes found within the cell; SOD1 (CuZn) is located in cytoplasm whilst SOD2 (Mn) is found in the mitochondria. Ectopic expression of either SOD1 or SOD2 gave no phenotype on their own (Fig 2D–2F) but were able to obviously suppress the Egr/TNFα eye (Fig 2G–2I). This suppression of the Egr/TNFα small eye phenotype was consistently observed in all progeny and supports a role for ROS in these Egr/TNFα-mediated phenotypes.

### WWOX remains cytoplasmically localised in response to ectopic Egr/TNFα expression

**Fig 3:**
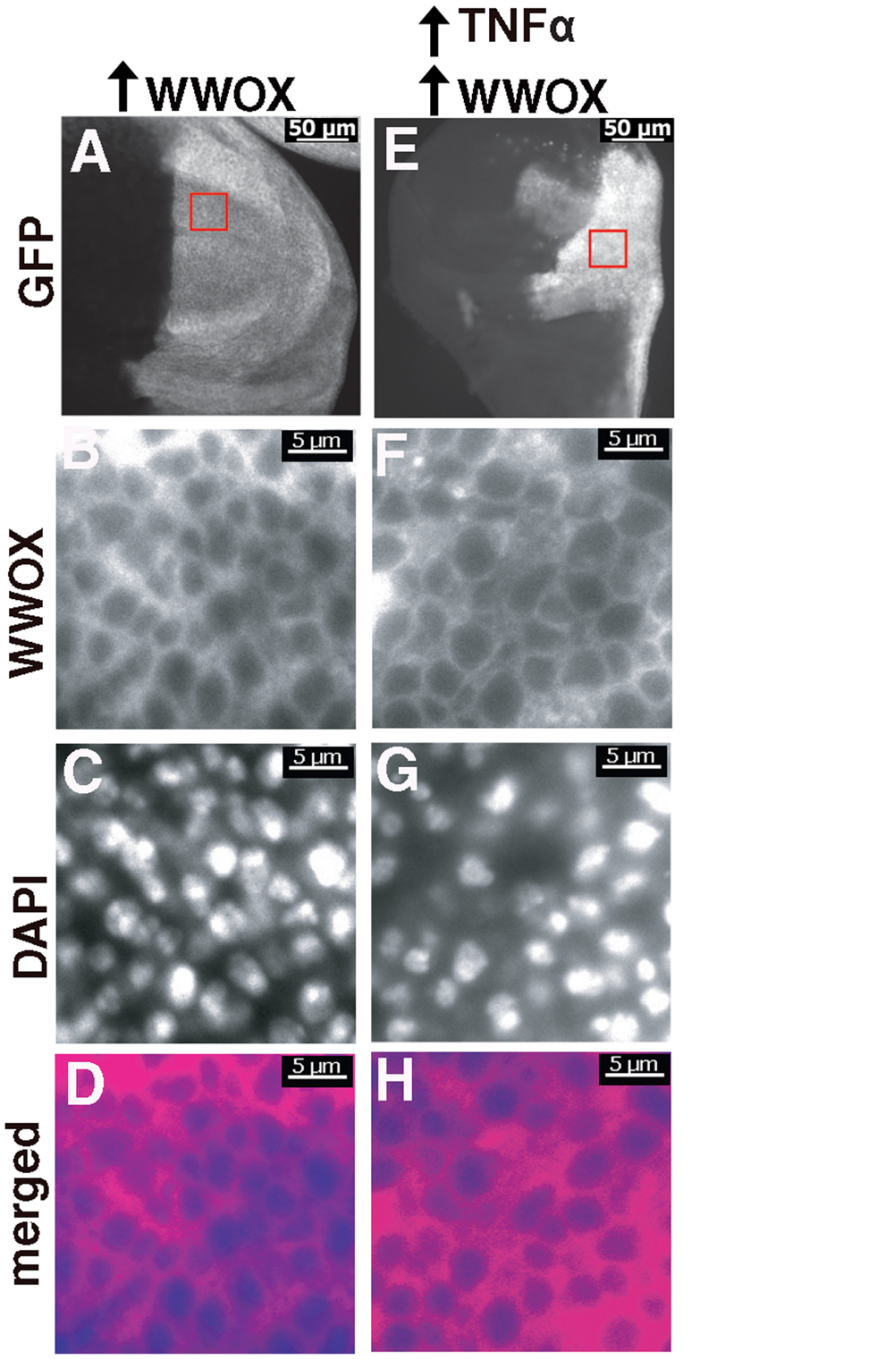
Ectopic Egr/TNFα has no obvious effect on cytoplasmically localized WWOX. **(A)** Ectopic expression of GFP and WWOX in the posterior portion of wing discs of wandering third instar larvae *(hh>GFP>WWOX)*, GFP shows the region of ectopic expression. **(B)** WWOX staining reveals expression localized to areas complementary to the DAPI stained nuclei shown in **(C)** with the merged image shown in **(D)**. **(E-H)** Ectopic expression of WWOX in the presence of ectopic Egr/TNFα *(hh>GFP>WWOX>egr* ^*+w*^ *)* also results in cytoplasmic localisation of WWOX. Nuclei/DAPI staining shown in blue and WWOX staining in magenta. Boxed regions shown in A and E correspond to regions that are enlarged in B-D and F-H respectively.

Nuclear localisation of WWOX has been reported to be necessary for the cell death promoting functions of WWOX in mammalian cells [42]. Although endogenous levels of WWOX are too low to be detected in *D*. *melanogaster*, we have previously shown cytoplasmic localization of ectopically expressed WWOX during embryonic development [49]. Here, we also determined the localisation of ectopically expressed WWOX in differentiated cells of the developing eye disc. *GMR-GAL4* was used to ectopically express WWOX in all cells posterior to the morphogenetic furrow. WWOX expression can be visualised in cytoplasmic regions surrounding the DAPI stained clusters of nuclei from photoreceptor cells (S3 Fig). A similar pattern of cytoplasmic WWOX expression was observed in the presence of ectopic Egr/TNFα expression (S3 Fig). Thus we observed no alteration to ectopic WWOX localisation in response to Egr/TNFα *in vivo*. Given the small size and complex organisation of cells in the developing eye disc, the effect of ectopic Egr/TNFα expression on the localisation of WWOX was also determined in cells in the posterior compartment of the wing disc using *hh-GAL4*. Co-expression of GFP allowed for the positive identification of cells in the region of ectopic expression. Ectopic WWOX alone resulted in cytoplasmic staining with WWOX detected in regions surrounding the DAPI stained nuclei throughout the posterior half of the disc (Fig 3A–3D). In the presence of ectopic Egr/TNFα expression, the wing discs are smaller and there is significant disruption to the region of the disc marked by GFP expression (Fig 3E). Closer examination of cells located in the posterior region of the disc showed that ectopic WWOX remains clearly cytoplasmic as staining was observed complementary to the DAPI stained nuclei (Fig 3F–3H). Thus there was no evidence *in vivo* for nuclear localisation of detectable levels of ectopic WWOX in response to Egr/TNFα expression in eye or wing imaginal discs.

### Ectopic Egr/TNFα alone promotes apoptosis and disrupts cellular patterning in wing discs

**Fig 4:**
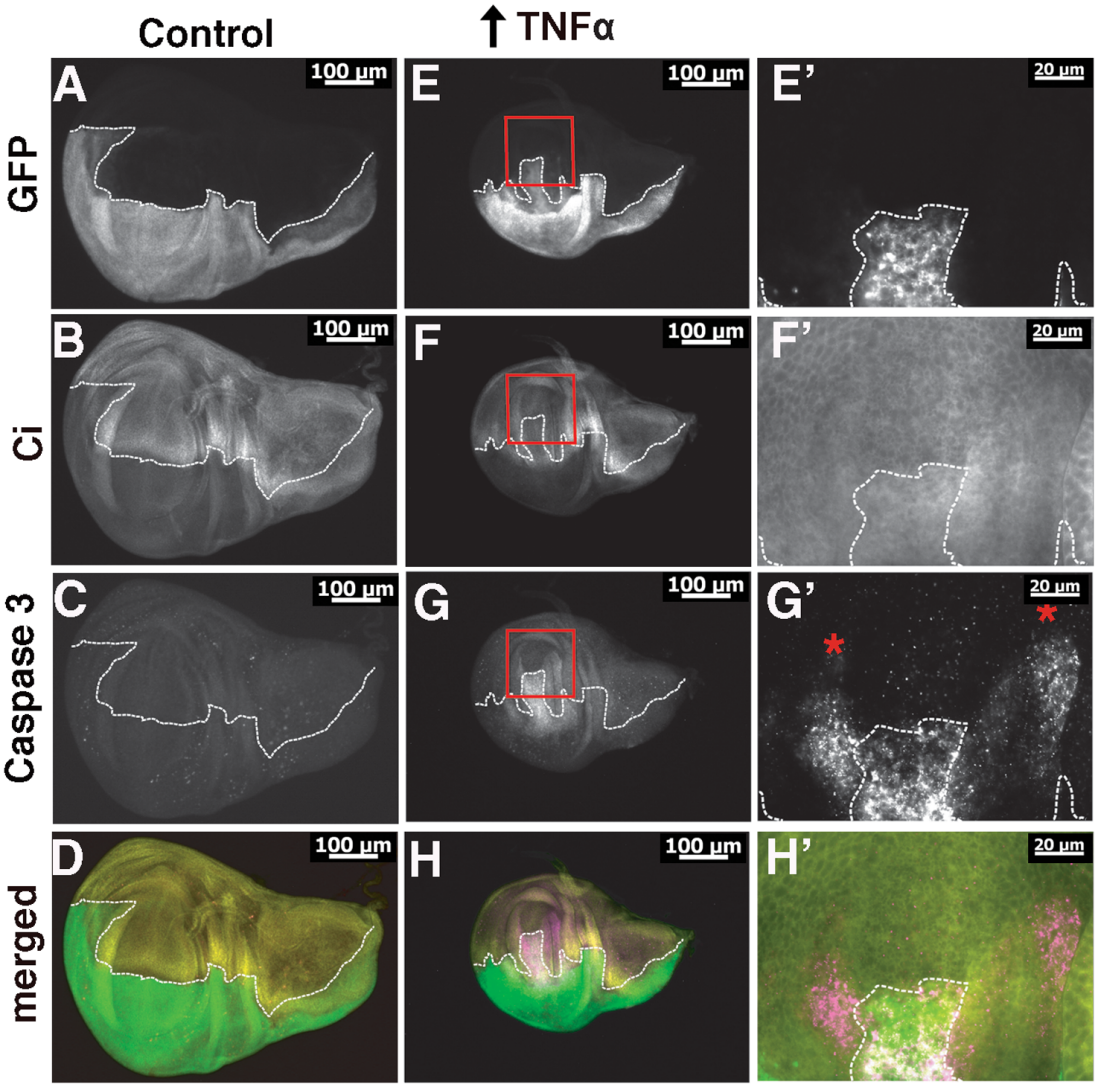
Qualitative effects of ectopic Egr/TNFα and increased apoptosis in the larval imaginal wing disc. **(A)** Control disc showing ectopic expression in the posterior region of wing discs using *hh-GAL4* visualized by co-expression of GFP. **(B)** Ci staining of control discs show staining of the anterior compartment and is complementary to the region of GFP expression. **(C)** Caspase 3 staining revealed low levels of apoptosis in control imaginal wing discs. **(D)** Merged image where GFP is green, Caspase 3 staining is magenta and Ci is yellow. **(E-E’)** Ectopic expression of Eiger/TNFα resulted in a significant decrease in disc size and disruption to the pattern of GFP expression with punctate staining in the central wing pouch region. **(F-F’)** Staining of the anterior compartment with Ci reveals expression beyond the boundary and overlapping with the region of GFP expression. **(G-G’)** Caspase 3 staining reveals high levels of staining in the central wing pouch region and in two distinct regions extending towards to anterior portion of the disc (indicated with the red asterisks). **(H-H’)** Merged image (GFP is green, Caspase 3 is magenta and Ci is yellow). In all images the dotted line outlines the regions of GFP expression corresponding to the posterior region of the discs. Red boxes indicate the regions that are enlarged in E’-H’.

Ectopic expression of Egr/TNFα alone in the posterior region of wing discs resulted in a significant decrease in tissue size and disruption to compartment boundaries as visualised by GFP expression (Fig 4E). In particular there is posterior GFP expression extending into the central wing pouch region of the disc (Fig 4E’). In order to determine the identity of these cells, Cubitis interruptus (Ci) staining was used as a marker of cells specific to the anterior portion of the wing disc. In control discs the region corresponding to Ci staining is complementary to the GFP expression pattern in the posterior region under control of *hh-GAL4* thus defining the boundary of these distinct cell types (Fig 4B). However, in response to ectopic Egr/TNFα expression in the posterior region, there is now a region of Ci positive anterior cells overlapping with the GFP positive posterior cells in the central wing pouch region (Fig 4F and 4F’). Thus ectopic expression of Egr/TNFα has resulted in disruption to normal patterning of the wing disc cells such that there is no longer a clear distinction between cells from the Ci staining portion of the disc (i.e. the wild-type cells from the anterior region) and GFP positive cells from the posterior part of the disc (i.e. cells ectopically expressing Egr/TNFα).

Furthermore, the GFP expression observed in this region of overlap was punctate in appearance suggestive of increased cell death (Fig 4E’). To assess the cell death we examined immunostaining for cleaved Caspase 3 [50]. Whilst negligible levels of Caspase 3 staining were observed in control discs (Fig 4C), increased levels were observed in the central wing pouch region of discs ectopically expressing Egr/TNFα (Fig 4G and 4G’). In addition, Caspase 3 staining was found to extend beyond the GFP region of the wing pouch in two distinct regions (Fig 4G’, asterisks). Similar localisation of Caspase 3 staining to these two regions has previously been reported following ectopic expression of Hid or Src64B, together with the apoptosis inhibitor P35 in the posterior region of developing wing discs [51–52]. The extremities of these regions have previously been shown to contain cells undergoing a process of Apoptosis-induced Apoptosis (AiA) with Egr/TNFα shown to be required for the death signal [52]. Closer examination of Cell Rox staining (Fig 2B) also revealed increased ROS corresponding to these two distinct regions. These results confirm that over-expression of a low level of Egr/TNFα in the posterior compartment is sufficient to induce ROS and cell death in anterior regions, consistent with a key role of Egr /TNFα as an activating signal for AiA.

### WWOX modifies Caspase 3 staining in response to ectopic Egr/TNFα

**Fig 5:**
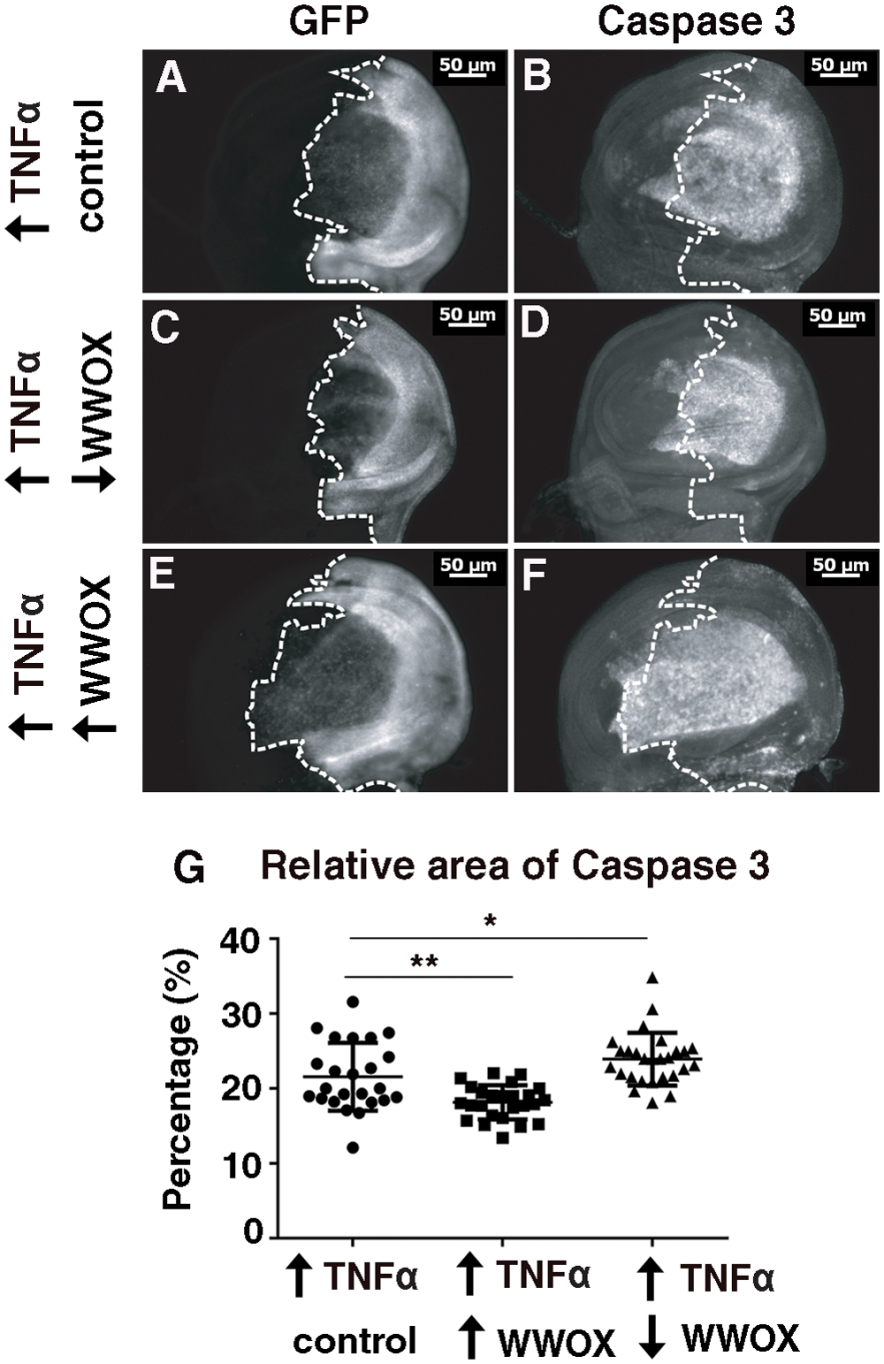
WWOX modifies Caspase3 staining in wing pouch in response to ectopic Egr/TNFα. **(A)** Ectopic expression of GFP and Eiger/TNFα with *hh-GAL4* in the posterior portion of wing discs of wandering third instar larvae, GFP showing the regions of ectopic expression. **(B)** Caspase 3 staining reveals high levels of apoptosis in the central wing pouch region as well as in two distinct regions extending towards the anterior. **(C)** Decreased WWOX expression results in a decrease in area of GFP expression. **(D)** Decreased WWOX expression results in a decreased region of Caspase 3 staining. **(E)** Increased WWOX expression results in an increase in area of GFP expression. **(F)** Increased WWOX expression results in a increased region of Caspase 3 staining. **(G)** Quantification of the area of Caspase 3 staining as a proportion of the area of the whole disc for individual wing discs of each genotype. Significance indicated by * = p<0.05, ** = p<0.005.

Since WWOX has been shown to modify adult eye phenotypes resulting from ectopic over-expression of Egr/TNFα, we determined whether WWOX was also able to regulate the increased region of Caspase 3 staining and consequent disruption to the patterning induced by ectopic expression of Egr/TNFα in wing discs. Decreased WWOX activity together with ectopic expression of Egr/TNFα in the posterior portion of the disc resulted in a decrease in the relative area of Caspase 3 staining (Fig 5C, 5D and 5G). Conversely, increased WWOX expression increased the relative area of Caspase 3 staining (Fig 5E–5G). Thus we have shown that WWOX activity is required for, and can contribute to, cell death in Egr/TNFα expressing cells via regulation of Caspase 3 activity. The outcome of this interaction at the interface between wild-type cells in the anterior and the posterior Egr/TNFα expressing cells is suggestive of competitive interactions between these two cell types.

### Requirement for WWOX tumor suppressor activity *in vivo*

**Fig 6:**
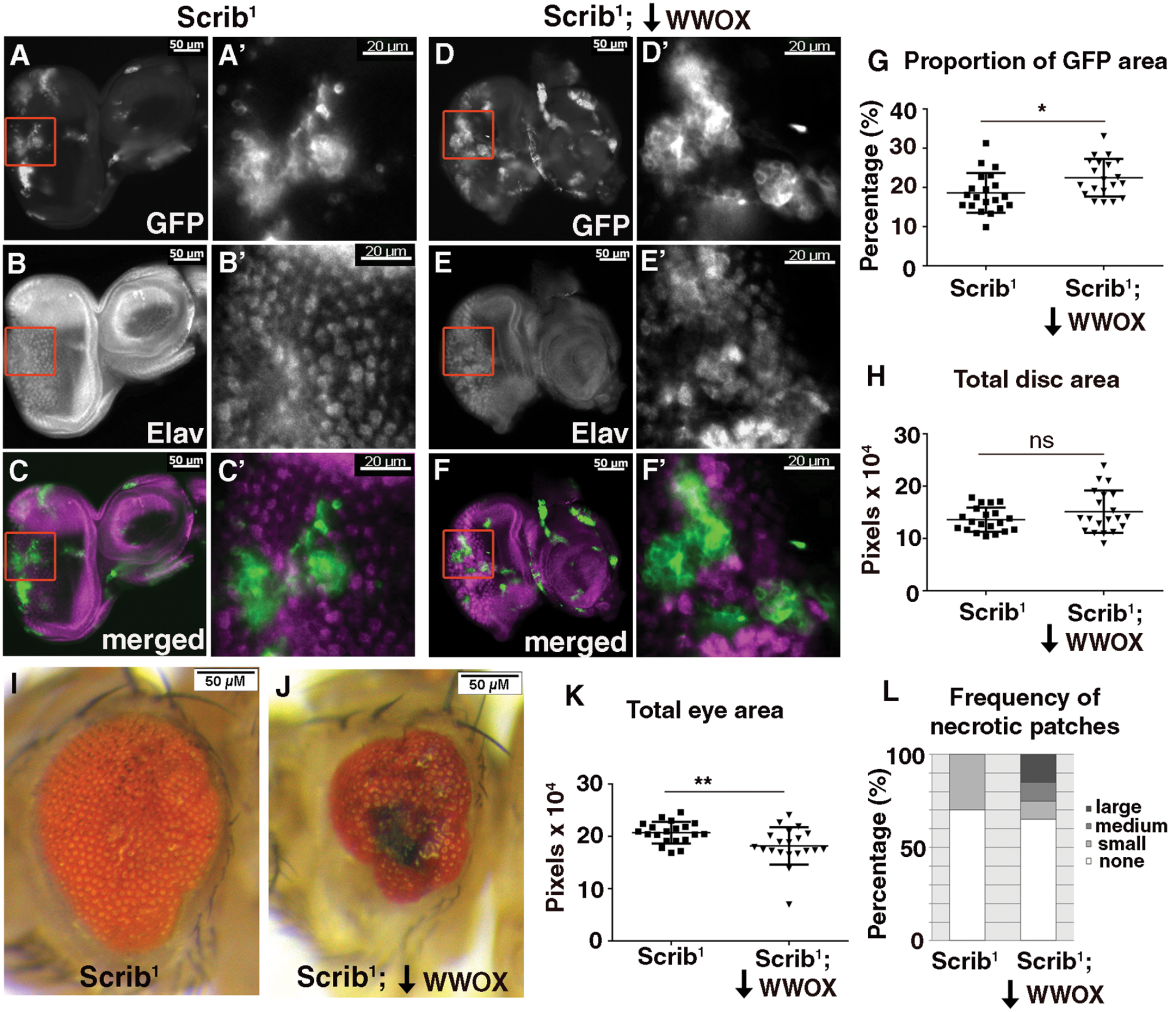
WWOX is required for elimination of *Scribbled (Scrib)* mutant clones. **(A-A’)** Clones of cells mutant for *Scrib* generated in the eye using the MARCM system are positively labelled with GFP. **(B-B’)** Elav staining reveals absence of differentiated photoreceptors within a portion of *Scrib* mutant clones. **(C-C’)** Merged image showing GFP in green and Elav in magenta. **(D-D’)** GFP expressing *Scrib* mutant clones with decreased WWOX expression *(Scrib* ^*1*^; *WWOX-IR)*. **(E-E’)** Elav staining reveals absence of differentiated photoreceptors within a portion of the *Scrib* ^*1*^;*WWOX-IR* mutant clones. **(F-F’)** Merged image showing GFP in green and Elav in magenta. Red boxes indicate the regions that are enlarged in D’-F’. **(G)** Quantification of the proportion of GFP expressing cells showed a significant increase when WWOX expression was decreased in the *Scrib* mutant clonal tissue compared to the *Scrib* mutant clones alone. **(H)** Quantification of total area of the eye disc containing *Scrib* clones with and without WWOX knockdown revealed no significant difference. **(I)** Clones of cells mutant for *Scrib* generated in the eye using the MARCM system result in a mild adult rough eye phenotype. **(J)** Decreased WWOX expression in the *Scrib* mutant clones gave a stronger phenotype with a decreased eye size, significant disruption to ommatidial patterning and the presence of necrotic lesions. **(K)** Quantification of the overall size of the adult eyes showed a significant decrease when WWOX levels were knocked down in *Scrib* mutant clones. **(L)** Quantification of percentage of adult fly eye showing necrotic spots of different sizes: Small (550–3000 pixels), Medium (3000–5500 pixels) or Large (>5500 pixels). Genotypes used in these experiments: *Scrib* ^*1*^ = *(ey-FLP1*, *UAS-mCD8-GFP;;tub-GAL4 FRT82B tub-GAL80/ FRT82B*, *Scrib* ^1^ *)*, *Scrib* ^*1*^;*WWOX-IR* = *(ey-FLP1*, *UAS-mCD8-GFP; UAS-WWOX-IR#2/+;tub-GAL4 FRT82B tub-GAL80/ FRT82B*, *Scrib* ^1^ *)*. Significance indicated by * = p<0.05 and ** = p<0.005, ns = not significant.

Competition occurs between cell types that are genetically distinct such that one has a competitive advantage over the other. In order to determine whether decreased WWOX impacts on the ability of tumorigenic cells to compete with non-tumorigenic / normal cells we utilized the well characterised system of mitotic clones of the cell polarity regulator Scribbled (*Scrib)*. Epithelial tissues in *D*. *melanogaster* where all cells are mutant for *Scrib* will overgrow and give rise to tumours [53]. However tumorigenic clones of *Scrib* mutant cells that are surrounded by wild-type cells will be eliminated [46, 54]. Clones of *Scrib* mutant cells generated in this way using the *M*osaic *A*nalysis with a *R*epressible *C*ell *M*arker (MARCM) system are positively labelled with GFP expression [55]. Many cells of the randomly generated mutant clones are eliminated however this process is not complete and some remain and can be visualized by patches of GFP positive cells in developing eye discs (Fig 6A and 6A’). These cells also correspond to regions of disruption to the normal pattern of differentiation as visualised by Elav staining during larval development (Fig 6B, 6B’, 6C and 6C’). When WWOX levels were reduced within these tumorigenic clones, an increase in the proportion of disc area with GFP positive cells was observed despite no change to overall disc size (Fig 6D, 6D’, 6G and 6H). These GFP positive cells were also found to correspond to regions disrupted in their differentiation as visualised with Elav (Fig 6E, 6E’, 6F and 6F’). Thus a decrease of WWOX within the clones of tumorigenic cells results in a mild but significant increase in their ability to compete, observed as a decrease in their effective elimination during this larval stage.

These tumorigenic *Scrib* clones persist throughout development and differentiation of eye tissue and result in mild adult eye phenotypes characterised by patches of roughness and disruption to ommatidial patterning (Fig 6I). This phenotype is enhanced when WWOX is decreased by RNAi knockdown within cells of the *Scrib* mutant clones where eyes consistently showed a significant decrease in size, as well as an increase in the frequency of black necrotic lesions of increased size on the surface of the adult eye (Fig 6J–6L). This enhanced phenotype also corresponds to an observed decrease in overall viability with flies with decreased WWOX expression in *Scrib* mutant clones showing a survival rate of 31.9% of that expected compared to 74.1% for flies with the *Scrib* mutant clones alone (**p = 0.0016). A decrease in adult viability (or increase in pupal lethality) has previously been reported as an indication of reduced elimination of *Scrib* mutant clones in other genetic backgrounds [45–46, 56]. Thus we have demonstrated a cell autonomous contribution from WWOX for the elimination of tumorigenic cells in a whole animal model system. Similar effects on adult eye development were obtained when *Scrib* mutant clones were generated in eye discs where the whole animal had reduced WWOX function (heterozygous for either of two independent alleles of *WWOX*) or where WWOX function is completely removed (trans-heterozygous for independent *WWOX* alleles) (S4 Fig). Together these findings confirm that there is a decrease in the effectiveness of the process whereby tumorigenic *Scrib* mutant clones are eliminated when WWOX activity is reduced either exclusively within cells comprising the mutant clones or when WWOX activity is reduced or completely removed from all cells of the animal. Although mild effects were observed during developmental stages they resulted in more significant outcomes at the end of differentiation.

## Discussion

The *WWOX* gene spanning *FRA16D* has previously been shown to have a variety of *in vitro* contributions to known cell death pathways in different mammalian cell lines, however it is unclear how these translate into a role *in vivo*, particularly in relation to the ability of WWOX to act as a tumor suppressor. We have therefore utilized a well-characterized *D*. *melanogaster* model of cell-cell competition to investigate an *in vivo* role for WWOX in the process of elimination of cancerous cells.

Significantly we determined an *in vivo* contribution by WWOX to the process whereby clones of epithelial cells carrying tumorigenic mutations are eliminated by the surrounding wild-type cells. The outcomes of competitive cell interactions in this way are essential contributing factors to the development of tumors *in vivo* [57]. We report here that reduction, or absence, of WWOX activity specifically in the tumorigenic cells decreased the effectiveness of this elimination process. Although this cell autonomous requirement for WWOX activity resulted in relatively mild effects on GFP expressing mutant cells in the eye imaginal discs, much more striking effects were evident at later stages. Generation of *Scrib* mutant clones in this way is analogous to the accumulation of mutations in cells that can gain a competitive advantage over the surrounding wild-type (non-mutant) cells and ultimately give rise to human cancers. Thus, our results show that endogenous WWOX plays a significant *in vivo* role in the process whereby mutation-bearing cells are eliminated. Together these results represent a plausible mechanism for low WWOX levels contributing to poor prognosis in various cancers [21–23,58].

We have also utilised *D*. *melanogaster* models to dissect the cell death pathways to which WWOX contributes *in vivo*. In mouse L929 cells, an ectopic increase in WWOX was found to enhance TNFα-mediated cell death [42]. Consistent with this observation, altered WWOX levels modulate the phenotype obtained from ectopic expression of Egr/TNFα in the eye of *D*. *melanogaster*. WWOX was also previously shown to be an essential component of p53-mediated apoptosis in NIH3T3 cells [42], however no impact of altered WWOX levels was observed on the *D*. *melanogaster* eye phenotype from ectopic expression of *p53*. Similarly, no WWOX-mediated alteration of the *D*. *melanogaster* eye phenotype from ectopic expression of *hid* was observed herein, although others have reported a mild effect with further reduced WWOX levels on ectopic *hid* expression in the *D*. *melanogaster* eye [59]. Together these data are consistent with WWOX having a conserved, biologically significant role to play in the cell death mediated by Egr/TNFα.

*In vitro* nuclear localisation of pro-apoptotic WWOX was reported in L929 cells [42] as well as in MC7 cells in response to DNA damage [60]. However, we found no *in vivo* evidence for nuclear localization of ectopic WWOX in the presence of ectopic TNFα expression, indicating that the tumor suppressive functions may not be at the level of detection or alternatively they may be mediated through cytoplasmic WWOX functions. Conflicting reports appear in the literature for the location of WWOX protein to various cytoplasmically localised organelles including Golgi and mitochondria [16, 49]. Thus the localisation of WWOX may vary in different cell types and in response to different cellular stressors.

**Fig 7:**
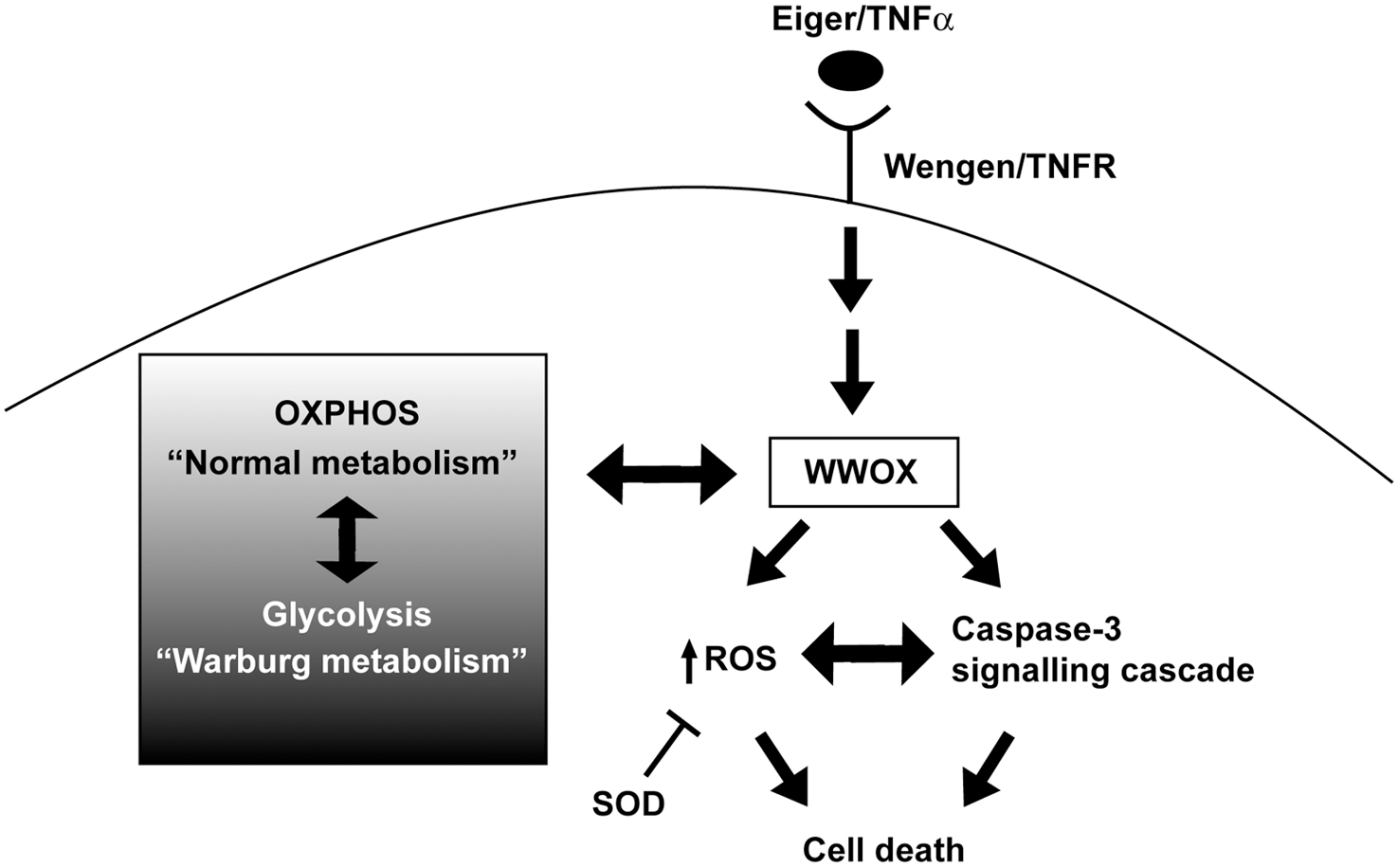
Model for the conserved role of WWOX in TNFα-mediated apoptosis *in vivo* in *D*. *melanogaster*. The function of WWOX in promoting cell death in response to Egr/TNFα signalling (through Wengen/TNFR) is mediated by ROS and Caspase 3. This can be modulated though expression of SOD enzymes that act to limit cellular levels of ROS and is also responsive to the metabolic status of cells.

We observed no phenotypic effect with ectopic expression of WWOX alone, thus the cell death promoting effects of WWOX may require that WWOX be activated or modified in some way (e.g. phosphorylation) and may only become effective *in vivo* once cells are under some type of stress. Reactive oxygen species (ROS) are known to be principle effector molecules of TNFα-mediated cell death [45]. We have previously shown ectopic expression of WWOX gives high levels of ROS whilst reduced levels of WWOX show decreased ROS in developing *D*. *melanogaster* larvae [30]. Therefore, a likely mechanism by which WWOX contributes to the Egr/TNFα-mediated cell death pathway is via its regulation of ROS (Fig 7). At least to some extent, this occurs through regulation of the subset of ROS that are also responsive to enzymes of the superoxide dismutase (SOD) family and we have previously shown alterations in isoforms of SOD1 in WWOX mutant flies as well as genetic interactions between WWOX and SOD1 [30]. However the role for WWOX in the regulation of ROS levels may occur in a context dependent manner given the opposing effects reported for altered ROS in response to modified WWOX expression [61–62]. In addition, alterations to ROS levels would occur as a consequence of cancer cells shifting their metabolism from oxidative phosphorylation to a more glycolytic Warburg-based metabolism and we have previously shown that WWOX is both responsive to, and contributes to aerobic metabolism [30–31]. The protein products of other Common Fragile Site-associated genes; *Fragile histidine triad* (*FHIT*) at *FRA3B* and *Parkin* at *FRA6E* have also previously been shown to act as regulators of ROS [63–65]. Thus these genes may act together to maintain genome integrity under conditions of heightened oxidative stress, potentially arising from alterations to cellular aerobic metabolism known to be associated with cancer.

## Materials and Methods

### Fly lines and crosses

*w* ^*1118*^, *UAS-Dmp53* [66], *GMR-GAL4*, *GMR-hid* [67], *hh-GAL4* [68–69], UAS-SOD1 and UAS-SOD2 [70] were provided by Bloomington Stock Centre. *UAS-TNFR-IR* (v9152), *UAS-WWOX-IR* ^*#1*^ (v22536), and *UAS-WWOX-IR* ^*#2*^ (v108350) were obtained from Vienna Drosophila RNAi Center. Successful knockdown of WWOX mRNA in each of these lines has previously been shown to be effective by quantitative real-time PCR (30, 61). Ectopic Eiger/TNFα expression stock (*UAS-egr* ^*+w*^) was kindly provided by Professor Miura [46]. *WWOX* ^*1*^, *WWOX* ^*2*^, *UAS-WWOX ORF* ^*#1*^ and *UAS-WWOX cDNA* ^*#1*^ have previously been described [49 and 61]. MARCMIII and *FRT82B*, *Scrib* ^*1*^ stocks were kindly provided by Helena Richardson. *D*. *melanogaster* stocks were maintained on fortified (F1) medium composed of 1% agar, 1% glucose, 6% fresh yeast, 9.3% molasses, 8.4% coarse semolina, 0.9% acid mix and 1.7% tegosept. All crosses were carried out at 25°C unless otherwise stated.

### Analyses of Adult Eyes

Photographs of exterior adult female *D*. *melanogaster* eyes were taken using an Olympus SZX7 microscope fitted with a SZX-AS aperture diaphragm unit. Images were captured using an Olympus ColourView IIIU Soft Imaging System camera and AnalysisRuler image acquisition software. Images prepared using Adobe Photoshop CS version 8.0. The anterior of eye is positioned to the left of all images. For determination of adult eye sizes the outline of ten different randomly selected eye photos were traced using ImageJ and total area (in pixels) for each image was measured. Results for each experiment were graphed as scatterplot and statistical analyses (T-test analyses and One Way ANOVA) performed in GraphPad Prism.

### Clonal analyses

Mitotic clones were generated for analyses using the MARCM III system, by crossing *ey-FLP1*, *UAS-mCD8-GFP;;tub-GAL4 FRT82B tub-GAL80/TM6B* flies to those carrying either a *WWOX* mutant allele or *WWOX* ^RNAi^ transgene together with *FRT82B*, *Scrib* ^1^. Timed lays were carried out for all eye disc analyses. Third instar wandering larvae were dissected in PBS and fixed with 4% formaldehyde before mounting in glycerol to visualise GFP expression (GFP indicative of clones and a minimum n = 20 eye discs were analysed per genotype). Significant disruption to eye disc morphology was observed in 13/52 pairs of the *Scrib* ^*1*^ clones and 31/50 pairs *Scrib* ^*1*^;*WWOX-IR* clones and these were not included in these analyses. The size of the whole eye disc and area of GFP clones were quantified using Image J. The clonal area was calculated as a percentage of the total size of the eye imaginal disc and the averaged results were graphed as a scatterplot. T-test analyses were performed using GraphPad Prism. For determination of necrotic spots, the area of the black regions on the surface of the adult eyes were measured using ImageJ and divided into/scored as different categories based on size; small (550–3000 pixels), medium (3000–5500 pixels) or large (>5500 pixels). The percentage of eyes in each category was calculated and graphed using Microsoft Excel. For the viability assays, the overall number of adult progeny that eclosed from pupae were scored and the ratio of non TM6B:TM6B progeny were recorded for each cross, as described previously (30). The survival rate is presented as a percentage of the expected ratio of progeny. Statistical analyses were performed using the chi-square test with p = 0.05 as cut off value for significance using GraphPad Prism.

### Western blot analyses

30 female adult flies (0–1 day old) per sample were collected and Western blot analyses were performed as previously described (49). Primary antibodies used were anti-C-DmWWOX antibody (1:1000) (49) and mouse monoclonal anti-β-tubulin antibody (1:2000, Sigma). Secondary antibodies used were Anti-Rabbit DyLight 649 antibody (1:2500, Vector Laboratories) and anti-mouse-Cy3antibody (1:200, Jackson Laboratories).

### Immunohistochemistry

Wing discs or eye imaginal discs were dissected from late third instar larvae in 1x phosphate buffered saline (PBS) and fixed in 3.7% formaldehyde for 20 minutes. Discs were then washed three times with PBST (1xPBS + 0.3% Triton-X-100) for 20 minutes and blocked with PBSTF (1xPBS containing 5% fetal calf serum) for 90 minutes, followed by incubation of primary antibody overnight at 4°C. Anti-C-DmWWOX antibody (1:100 (52), anti-cleaved Caspase 3 antibody (1:100, Cell Signaling), anti-Elav 9F8A9 (1:10, Developmental Studies Hybridoma Bank) and anti-Ci 2A1 (1:100, Developmental Studies Hybridoma Bank) were used as primary antibodies. Discs were washed with PBST three times for 20 minutes and blocked with PBSTF for 30 minutes, followed by incubation of secondary antibody in the dark at room temperature for 2 hours. Secondary antibodies used were Anti-Rabbit DyLight 649 antibody (1:100, Vector Laboratories) and Anti-Rat rhodamine antibody (1:100). Discs were then washed three times with PBST for 20 minutes before incubation of DAPI (1:1000) for five minutes at room temperature and mounting in 80% glycerol. Relative areas of Caspase 3 staining were quantified in Image J and analysed in GraphPad Prism.

### Cell ROS Assay

Reactive oxygen species (ROS) in third instar wing disc were detected using the fluorogenic probe CellRox (Life Technologies) as described previously [70].

## Supporting Information

S1 Fig Altered WWOX modifies ectopic Egr/TNFα-mediated eye phenotype. **(A)** Ectopic expression of Egr/TNFα (GMR>*egr* ^*+w*^ *>+*) results in a decrease in eye size and disruption to ommatidial patterning. **(B)** Decreased expression of WWOX by RNAi knockdown (*GMR>egr* ^*+w*^ *>WWOX-IR* ^*#2*^) resulted in suppression of the rough eye phenotype. **(C)** Decreased expression of WWOX by heterozygous null allele (*GMR>egr* ^*+w*^ *>WWOX* ^*1*^ */+*) resulted in suppression of the rough eye phenotype. **(D)** Decreased expression of WWOX by heterozygous insertion mutation allele (*GMR>egr* ^*+w*^ *>WWOX* ^*2*^ */+*) resulted in suppression of the rough eye phenotype. **(E)** Increased expression of WWOX (GMR>*egr* ^*+w*^ *>WWOX-ORF*) resulted an enhancement of the Egr/TNFα phenotype. **(F)** Quantification of increased eye size with independent heterozygous *WWOX* alleles (*GMR>egr* ^*+w*^ *>WWOX* ^*1*^ */+* and *GMR>egr* ^*+w*^ *>WWOX* ^*2*^ */+)*. **(G)** Increased expression of WWOX alone by ectopic expression of the ORF for WWOX (GMR>*WWOX-ORF*) resulted in no effect on development of the adult eye. **(H)** Western blot analysis and **(I)** quantification of the relative levels of WWOX protein expressed in each of the ectopic expression lines compared to a β-Tubulin control. **(J)** Western blot analysis and **(K)** quantification of WWOX protein expressed alone and together with Egr/TNFα compared to a β-Tubulin control.
(TIF)

S2 Fig Altered WWOX has no effect on ectopic p53 or Hid eye phenotypes. **(A)** Ectopic expression of Dmp53 in the developing eye *(GMR>DmP53>EV)* at 18°C in the adult eye results in a phenotype characterized by decrease in eye size and significant disruption to ommatidial patterning accompanied by loss of pigment and the presence of small necrotic lesions. **(B)** Decreased expression of WWOX by RNAi *(GMR>DmP53>WWOX-IR* ^*#1*^ *)* resulted in no significant modification. **(C)** Increased expression of WWOX *(GMR>DmP53>WWOX-cDNA)* also resulted in no significant modification. **(D)** Ectopic expression of head involution defective in the adult eye (GMR>*GMR-Hid>EV*) results in a very strong rough eye phenotype with reduction in eye size and almost complete loss of ommatidial structures. **(E)** Decreased expression of WWOX by RNAi *(GMR>GMR-Hid>WWOX-IR* ^*#1*^ *)* resulted in no significant modification. **(F)** Increased expression of WWOX *(GMR>GMR-Hid>WWOX-cDNA)* also resulted in no significant modification.
(TIF)

S3 Fig Ectopic Egr/TNFα has no effect on the cytoplasmic localization of WWOX in the eye imaginal disc. **(A-D)** Ectopic expression of WWOX alone with *GMR-gal4* results in WWOX localisation to areas complementary to the DAPI stained nuclei of eye-imaginal discs of wandering third instar larvae. **(E-H)** Ectopic expression of WWOX with *GMR-gal4* in the presence of ectopic Egr/TNFα expression also results in WWOX localisation to areas complementary to the DAPI stained nuclei of eye-imaginal discs of wandering third instar larvae.
(TIF)

S4 Fig WWOX is required for elimination of *Scribbled (Scrib)* mutant clones in adult eyes. **(A)** Clones of cells mutant for *Scrib* generated in the eye using the MARCM system result in a mild adult rough eye phenotype. **(B)** Decreased WWOX expression throughout the whole animal (*Scrib* ^*1*^; *WWOX* ^*1*^ */+)* resulted in a stronger phenotype with a decreased eye size, significant disruption to ommatidial patterning and the presence of some necrotic lesions. **(C)** Complete absence of WWOX throughout the whole animal (*Scrib* ^*1*^; *WWOX* ^*1*^ */ WWOX* ^*2*^ *)* resulted in a phenotype with a decreased eye size, significant disruption to ommatidial patterning and the presence of large necrotic lesions. Genotypes used: *Scrib* ^*1*^ *(ey-FLP1*, *UAS-mCD8-GFP; +/+;tub-GAL4 FRT82B tub-GAL80/ FRT82B scrib* ^1^ *)*, *Scrib* ^*1*^;*WWOX* ^*-/+*^ *(ey-FLP1*, *UAS-mCD8-GFP; WWOX* ^*1*^ */+;tub-GAL4 FRT82B tub-GAL80/ FRT82B scrib* ^1^ *)*, *Scrib* ^*1*^; *WWOX* ^*-/-*^ = *(ey-FLP1*, *UAS-mCD8-GFP; WWOX* ^*1*^ */ WWOX* ^*2*^;*tub-GAL4 FRT82B tub-GAL80/ FRT82B scrib* ^1^). Adults carrying *WWOX* mutations were generated by crossing *ey-FLP1*, *UAS-mCD8-GFP;;tub-GAL4 FRT82B tub-GAL80/TM6B* flies carrying either a *WWOX* mutant allele or *wild-type* second chromosome together with *FRT82B*, *Scrib* ^1^ carrying a *WWOX* mutant allele.
(TIF)

## References

[1] Hanahan D, Weinberg RA. Hallmarks of cancer: the next generation. Cell. 2011; 144: 646–674. 10.1016/j.cell.2011.02.013 21376230

[2] Yunis JJ, Soreng AL. Constitutive fragile sites and cancer. Science. 1984; 226: 1199–1204. 6239375 10.1126/science.6239375

[3] Glover TW, Arit MF, Casper AM, Durkin SG. Mechanisms of common fragile site instability. Hum Mol Genet. 2005; 14 Spec No. 2, R197–205. 16244318 10.1093/hmg/ddi265

[4] O'Keefe LV, Richards RI. Common chromosomal fragile sites and cancer: focus on FRA16D. Cancer Lett. 2006; 232: 37–47. 16242840 10.1016/j.canlet.2005.07.041

[5] Bignell GR, Greenman CD, Davies H, Butler AP, Edkins S, Andrews JM, et al Signatures of mutation and selection in the cancer genome. Nature. 2010; 463: 893–898. 10.1038/nature08768 20164919 PMC3145113

[6] Gao G, Smith DI. Very large common fragile site genes and their potential role in cancer development. Cell Mol Life Sci. 2014; 71: 4601–4615. 10.1007/s00018-014-1753-6 25300511 PMC11113612

[7] Mangelsdorf M, Reid K, Woollatt E, Dayan S, Eyre H, Finnis M, et al Chromosomal fragile site FRA16D and DNA instability in cancer. Cancer Res. 2000; 60:1683–1689. 10749140

[8] Ried K, Finnis M, Hobson L, Mangelsdorf M, Dayan S, Nancarrow JK, et al Common chromosomal fragile site FRA16D sequence: identification of the FOR gene spanning FRA16D and homozygous deletions and translocation breakpoints in cancer cells. Hum Mol Genet. 2000; 9:1651–1663. 10861292 10.1093/hmg/9.11.1651

[9] Schrock MS, Huebner K. WWOX: A fragile tumor suppressor. Exp Biol Med. (Maywood) 2014; pii: 1535370214561590. 10.1177/1535370214561590 PMC4471953 25538133

[10] Gardenswartz A, Aqeilan RI. WW domain-containing oxidoreductase's role in myriad cancers: clinical significance and future implications. Exp Biol Med (Maywood). 2014; 239: 253–263. 24510053 10.1177/1535370213519213

[11] Baryła I, Styczen-Binkowska E, Bednarek AK. Alteration of WWOX in human cancer, a clinical view. Experimental Biology and Medicine. 2015; 0: 1–10. 10.1177/1535370214561953 PMC4935223 25681467

[12] Yang L, Liu B, Huang B, Deng J, Li H, Yu B, et al A functional copy number variation in the WWOX gene is associated with lung cancer risk in Chinese. Hum Mol Genet. 2013; 22: 1886–1894. 10.1093/hmg/ddt019 23339925

[13] Yu K, Fan J, Ding X, Li C, Wang J, Xiang Y, et al Association study of a functional copy number variation in the WWOX gene with risk of gliomas among Chinese people. Int J Cancer. 2014; 10.1002/ijc.28815 24585490

[14] Ludes-Meyers J H, Kil H, Nuñez MI, Conti CJ, Parker-Thornburg J, Bedford MT, et al. WWOX hypomorphic mice display a higher incidence of B-cell lymphomas and develop testicular atrophy. Genes Chromosomes Cancer. 2007; 46: 1129–1136. 17823927 10.1002/gcc.20497 PMC4143238

[15] Aqeilan RI, Trapasso F, Hussain S, Costinean S, Marshall D, Pekarsky Y, et al Targeted deletion of WWOX reveals a tumor suppressor function. Proc Natl Acad Sci. USA 2007; 104: 3949–3954. 17360458 10.1073/pnas.0609783104 PMC1820689

[16] Bednarek AK, Keck-Waggoner CL, Daniel RL, Laflin KJ, Bergsagel PL, Kiguchi K, et al WWOX, the FRA16D gene, behaves as a suppressor of tumor growth. Cancer Res. 2001; 61: 8068–8073. 11719429

[17] Fabbri M, Iliopoulos D, Trapasso F, Aqeilan RI, Cimmino A, Zanesi N, et al WWOX gene restoration prevents lung cancer growth in vitro and in vivo. Proc Natl Acad Sci U S A. 2005; 102: 15611–15616. 16223882 10.1073/pnas.0505485102 PMC1266103

[18] Nakayama S, Semba S, Maeda N, Aqeilan RI Huebner K, Yokozaki H. Role of the WWOX gene, encompassing fragile region FRA16D, in suppression of pancreatic carcinoma cells. Cancer Sci. 2008; 99: 1370–1376. 10.1111/j.1349-7006.2008.00841.x 18460020 PMC11159152

[19] Iliopoulos D, Fabbri M, Druck T, Qin HR, Han SY, Huebner K. Inhibition of breast cancer cell growth in vitro and in vivo: effect of restoration of WWOX expression. Clin Cancer Res. 2007; 13: 268–274. 17200365 10.1158/1078-0432.CCR-06-2038

[20] Qin HR, Iliopoulos D, Semba S, Fabbri M, Druck T, Volinia S, et al A role for the WWOX gene in prostate cancer. Cancer Res. 2006; 66: 6477–6481. 16818616 10.1158/0008-5472.CAN-06-0956

[21] Pluciennik E, Kusinska R, Potemski P, Kubiak R, Kordek R, Bednarek AK. WWOX—the FRA16D cancer gene: expression correlation with breast cancer progression and prognosis. Eur J Surg Oncol. 2006; 32: 153–157 16360296 10.1016/j.ejso.2005.11.002

[22] Zelazowski MJ, Pluciennik E, Pasz-Walczak G, Potemski P, Kordek R, Bednarek AK. WWOX expression in colorectal cancer—a real-time quantitative RT-PCR study. Tumour Biol 2011; 32: 551–560. 10.1007/s13277-010-0150-5 21347750 PMC3093543

[23] Lin JT, Tzai TS, Liao CY, Wang JS, Wu TT, Wang HY, et al WWOX protein expression varies among RCC histotypes and downregulation of WWOX protein correlates with less-favorable prognosis in clear RCC. Ann Surg Oncol. 2013; 20: 193–199. 10.1245/s10434-012-2371-x 22555346

[24] Chang JY, He RY, Lin HP, Hsu LJ, Lai FJ, Hong Q, et al Signaling from membrane receptors to tumor suppressor WW domain-containing oxidoreductase. Exp Biol Med (Maywood). 2010; 235: 796–804. 20542955 10.1258/ebm.2010.009351

[25] Salah Z, Aqeilan R, Huebner K. WWOX gene and gene product: tumor suppression through specific protein interactions. Future Oncol. 2010 6:249–59. 10.2217/fon.09.152 20146584 PMC2832309

[26] Richards R, Choo A, Lee CS, Dayan S, O'Keefe L. WWOX, the chromosomal fragile site FRA16D spanning gene: its role in metabolism and contribution to cancer. Exp Biol & Med (Maywood). 2015; pii: 1535370214565990. 10.1177/1535370214565990 PMC4935231 25595186

[27] Aqeilan RI, Hagan JP, Aqeilan HA, Pichiorri F, Fong LY, Croce CM. Inactivation of the WWOX gene accelerates forestomach tumor progression in vivo. Cancer Res. 2007; 67: 5606–5610. 17575124 10.1158/0008-5472.CAN-07-1081 PMC2621009

[28] Ludes-Meyers JH, Kil H, Parker-Thornburg J, Kusewitt DF, Bedford MT, Aldaz CM. Generation and characterization of mice carrying a conditional allele of the WWOX tumor suppressor gene. PLoS ONE. 2009; 4: e7775 10.1371/journal.pone.0007775 19936220 PMC2777388

[29] Suzuki H, Katayama K, Takenaka M, Amakasu K, Saito K, Suzuki K. A spontaneous mutation of the WWOX gene and audiogenic seizures in rats with lethal dwarfism and epilepsy. Genes Brain Behav. 2009; 8: 650–660. 10.1111/j.1601-183X.2009.00502.x 19500159

[30] O'Keefe LV, Colella A, Dayan S, Chen Q, Choo A, Jacob R, et al. *Drosophila* orthologue of WWOX, the chromosomal fragile site FRA16D tumor suppressor gene, functions in aerobic metabolism and regulates reactive oxygen species. Hum Mol Genet 2011; 20: 497–509. 10.1093/hmg/ddq495 21075834 PMC3016910

[31] Dayan S, O’Keefe LV, Choo A, Richards RI. Common chromosomal fragile site FRA16D tumor suppressor WWOX gene expression and metabolic reprogramming in cells. Gene Chromosomes Cancer. 2013; 52: 823–831. 10.1002/gcc.22078 23765596

[32] Lo JY, Chou YT, Lai FJ, Hsu LJ. Regulation of cell signaling and apoptosis by tumor suppressor WWOX. Exp Biol Med (Maywood). 2015; pii: 1535370214566747. 10.1177/1535370214566747 PMC4935227 25595191

[33] Zhang H, Kong L, Cui Z, Du W, He Y, Yang Z, et al The WWOX gene inhibits the growth of U266 multiple myeloma cells by triggering the intrinsic apoptotic pathway. Int J Mol Med. 2014; 34: 804–809. 10.3892/ijmm.2014.1824 24968878

[34] Nowakowska M, Pospiech K, Lewandowska U, Piastowska-Ciesielska AW, Bednarek AK. Diverse effect of WWOX overexpression in HT29 and SW480 colon cancer cell lines. Tumor Biol. 2014; 35: 9291–9301. 10.1007/s13277-014-2196-2 PMC4190457 24938873

[35] Wei D, Zhang X, Zou H, Wang L, Fu B, Wu X, et al. WW domain containing oxidoreductase induces apoptosis in gallbladder-derived malignant cell by upregulating expression of P73 and PUMA. Tumor Biol. 2014; 35: 1539–1550. 10.1007/s13277-013-1213-1 24127039

[36] Qu J, Lu W, Li B, Lu C, Wan X. WWOX induces apoptosis and inhibits proliferation in cervical cancer and cell lines. Int J Mol Med. 2013; 31: 1139–1147. 10.3892/ijmm.2013.1314 23525362

[37] Cui Z, Lin D, Cheng F, Luo L, Kong L, Xu J, et al The role of the WWOX gene in leukemia and its mechanisms of action. Oncol Rep. 2013; 29: 2154–2162. 10.3892/or.2013.2361 23525648

[38] Chiang MF, Yeh ST, Liao HF, Chang NS, Chen YJ. Overexpression of WW domain-containing oxidoreductase WOX1 preferentially induces apoptosis in human glioblastoma cells harboring mutant p53. Biomed Pharmacother. 2012; 66: 433–438. 10.1016/j.biopha.2012.03.003 22898080

[39] Kosla K, Pluciennik E, Kurzyk A, Jesionek-Kupnicka D, Kordek R, Potemski P, et al Molecular analysis of WWOX expression correlation with proliferation and apoptosis in glioblastoma multiforme. J. Neurooncol. 2011; 101: 207–213. 10.1007/s11060-010-0254-1 20535528 PMC2996532

[40] Hu BS, Tan JW, Zhu GH, Wang DF, Zhou X, Sun ZQ. WWOX induces apoptosis and inhibits proliferation of human hepatoma cell line SMMC-7721. World J Gastroenterol. 2012; 18: 3020–3026. 10.3748/wjg.v18.i23.3020 22736928 PMC3380332

[41] Lai FJ, Cheng CL, Chen ST, Wu CH, Hsu LJ, Lee JY, et al. WOX1 is essential for UVB irradiation-induced apoptosis and down-regulated via translational blockade in UVB-induced cutaneous squamous cell carcinoma in vivo. Clin Cancer Res. 2005; 11: 5769–5777. 16115915 10.1158/1078-0432.CCR-04-2274

[42] Chang NS, Pratt N, Heath J, Schultz L, Sleve D, Carey GB, et al Hyaluronidase induction of a WW domain-containing oxidoreductase that enhances tumor necrosis factor cytotoxicity. J Biol. Chem. 2001; 276: 3361–3370. 11058590 10.1074/jbc.M007140200

[43] Igaki T, Kanda H, Yamamoto-Goto Y, Kanuka H, Kuranaga E, Aigaki T, et al Eiger, a TNF superfamily ligand that triggers the Drosophila JNK pathway. EMBO J. 2002; 21: 3009–3018. 12065414 10.1093/emboj/cdf306 PMC126061

[44] Moreno E, Yan M, Basler K. Evolution of TNF signaling mechanisms: JNK-dependent apoptosis triggered by Eiger, the Drosophila homolog of the TNF superfamily. Curr Biol. 2002; 12: 1263–1268. 12176339 10.1016/s0960-9822(02)00954-5

[45] Kanda H, Igaki T, Okano H, Miura M. Conserved metabolic energy production pathways govern Eiger/TNF-induced nonapoptotic cell death. Proc Natl Acad Sci U S A. 2011; 108: 18977–18982. 10.1073/pnas.1103242108 22065747 PMC3223446

[46] Igaki T, Pastor-Pareja JC, Aonuma H, Miura M, Xu T. Intrinsic tumor suppression and epithelial maintenance by endocytic activation of Eiger/TNF signaling in Drosophila. Dev Cell. 2009; 16: 458–465. 10.1016/j.devcel.2009.01.002 19289090 PMC2729686

[47] Kauppila S, Maaty WSA, Chen P, Tomar RS, Eby MT, Chapo J, et al Eiger and its receptor, Wengen, comprise a TNF-like system in *Drosophila* . Oncogene. 2003; 22: 4860–4867. 12894227 10.1038/sj.onc.1206715

[48] Kanda H, Igaki T, Kanuka H, Yagi T, Miura M. Wengen, a member of the Drosophila tumor necrosis factor receptor superfamily, is required for Eiger signaling. J Biol Chem. 2002; 277: 28372–28375. 12084706 10.1074/jbc.C200324200

[49] O'Keefe LV, Liu Y, Perkins A, Dayan S, Saint R, Richards RI. FRA16D common chromosomal fragile site oxido-reductase (FOR/WWOX) protects against the effects of ionizing radiation in Drosophila. Oncogene. 2005; 24: 6590–6596. 16007179 10.1038/sj.onc.1208806

[50] Denton D, Kumar S. Immunostaining using an antibody against active caspase-3 to detect apoptotic cells in Drosophila. Spring Harb Protoc. 2015; 10.1101/pdb.prot086215 26034309

[51] Pérez-Garijo A, Fuchs Y, Steller H. Apoptotic cells can induce non-autonomous apoptosis through the TNF pathway. Elife. 2013; 2:e01004 10.7554/eLife.01004 24066226 PMC3779319

[52] Fernández BG, Jezowska B, Janody F. Drosophila actin-Capping protein limits JNK activation by the Src proto-oncogene. Oncogene. 2014; 33: 2027–2039 10.1038/onc.2013.155 23644660

[53] Bilder D Epithelial polarity and proliferation control: links from the Drosophila neoplastic tumor suppressors. Genes Dev. 18 2004; 1909–1925. 15314019 10.1101/gad.1211604

[54] Ohsawa S, Sugimura K, Takino K, Xu T, Miyawaki A, Igaki T. Elimination of oncogenic neighbors by JNK-mediated engulfment in Drosophila. Dev Cell. 2011; 20: 315–328. 10.1016/j.devcel.2011.02.007 21397843

[55] Lee T, Luo L. Mosaic analysis with a repressible cell marker (MARCM) for Drosophila neural development. Trends Neurosci. 2001; 24: 251–254. 11311363 10.1016/s0166-2236(00)01791-4

[56] Brumby AM, Richardson HE. Scribbled mutants cooperate with oncogenic Ras and Notch to cause neoplastic overgrowth in Drosophila. EMBO J. 2003; 22: 5769–5779. 14592975 10.1093/emboj/cdg548 PMC275405

[57] Wagstaff L, Kolahgar G, Piddini E. Competitive cell interactions in cancer: a cellular tug of war. Trends Cell Biol. 2013; 23: 160–167. 10.1016/j.tcb.2012.11.002 23219382

[58] Park SW, Ludes-Meyer J, Zimonjic DB, Durkin ME, Popescu NC, Aldaz CM. Frequent downregulation and loss of WWOX gene expression in human hepatocellular carcinoma. Br J Cancer. 2004; 91: 753–759. 15266310 10.1038/sj.bjc.6602023 PMC2364795

[59] Schoenherr JA, Drennan JM, Martinez JS, Chikka MR, Hall MC, Chang HC, et al *Drosophila* Activated Cdc42 Kinase Has an Anti-Apoptotic Function. PLoS Genet. 2012; 8(5): e1002725 22615583 10.1371/journal.pgen.1002725 PMC3355085

[60] Abu-Odeh M, Salah Z, Herbel C, Hofmann TG, Aqeilan RI. WWOX, the common fragile site FRA16D gene product, regulates ATM activation and the DNA damage response. Proc Natl Acad Sci U S A. 2014 111:E4716–25. 10.1073/pnas.1409252111 25331887 PMC4226089

[61] Choo A, O’Keefe LV, Lee CS, Gregory SL, Shaukat Z, Lee CA, et al Tumour suppressor *WWOX* moderates the mitochondrial respiratory complex. *Genes*, *Chromosomes and Cancer* *(in press)*. 10.1002/gcc.22286 26390919

[62] Shaukat Z, Liu D, Choo A, Hussain R, O’Keefe L, Richards R, et al Chromosomal instability causes sensitivity to metabolic stress. Oncogene. 2014; 10.1038/onc.2014.344 25347746

[63] Trapasso F, Pichiorri F, Gaspari M, Palumbo T, Aqeilan RI, Gaudio E, et al Fhit interaction with ferredoxin reductase triggers generation of reactive oxygen species and apoptosis of cancer cells. J Biol Chem. 2008; 283: 13736–13744. 10.1074/jbc.M709062200 18319262 PMC2376222

[64] Karras JR, Paisie CA, Huebner K. Replicative stress and the FHIT gene: roles in tumor suppression, genome stability and prevention of carcinogenesis. Cancers. 2014; 6:1208–1219. 10.3390/cancers6021208 24901304 PMC4074825

[65] Palacino JJ, SAgi D, Goldberg MS, Krauss S, Motz C, Wacker M, et al Mitochondrial dysfunction and oxidative damage in parkin-deficient mice. J Biol Chem 2004; 279: 18614–18622. 14985362 10.1074/jbc.M401135200

[66] Ollmann M, Young LM, Di Como CJ, Karim F, Belvin M, Robertson S, et al Drosophila p53 is a structural and functional homolog of the tumor suppressor p53. Cell. 2000; 101: 91–101. 10778859 10.1016/S0092-8674(00)80626-1

[67] Grether ME, Abrams JM, Agapite J, White K, Steller H. The head involution defective gene of Drosophila melanogaster functions in programmed cell death. Genes Dev. 1995; 9: 1694–1708. 7622034 10.1101/gad.9.14.1694

[68] Evans CJ, Olson JM, Ngo KT, Kim E, Lee NE, Kuoy E, et al G-TRACE: rapid Gal4-based cell lineage analysis in Drosophila. Nat Methods. 2009; 6: 603–605. 10.1038/nmeth.1356 19633663 PMC2754220

[69] Ibrahim DM, Biehs B, Kornberg TB, Kiebes A. Microarray comparison of anterior and posterior Drosophila wing imaginal disc cells identifies novel wing disc. G3 (Bethesda). 2013; 3: 1353–1362. 23749451 10.1534/g3.113.006569 PMC3737175

[70] Anderson PR, Kirby K, Orr WC, Hilliker AJ, Phillips JP. Hydrogen peroxide scavenging resuces frataxin deficiency in a Drosophila model of Friedreich’s ataxia. Proc Natl Acad Sci U S A. 2008; 105: 611–616. 10.1073/pnas.0709691105 18184803 PMC2206584

