# Supplementary figures and images for: Tumor Suppressor WWOX Contributes to the Elimination of Tumorigenic Cells in Drosophila melanogaster
Source: PLoS One. 2015 Aug 24;10(8):e0136356. doi: 10.1371/journal.pone.0136356 (PMC4547717; doi:10.1371/journal.pone.0136356)

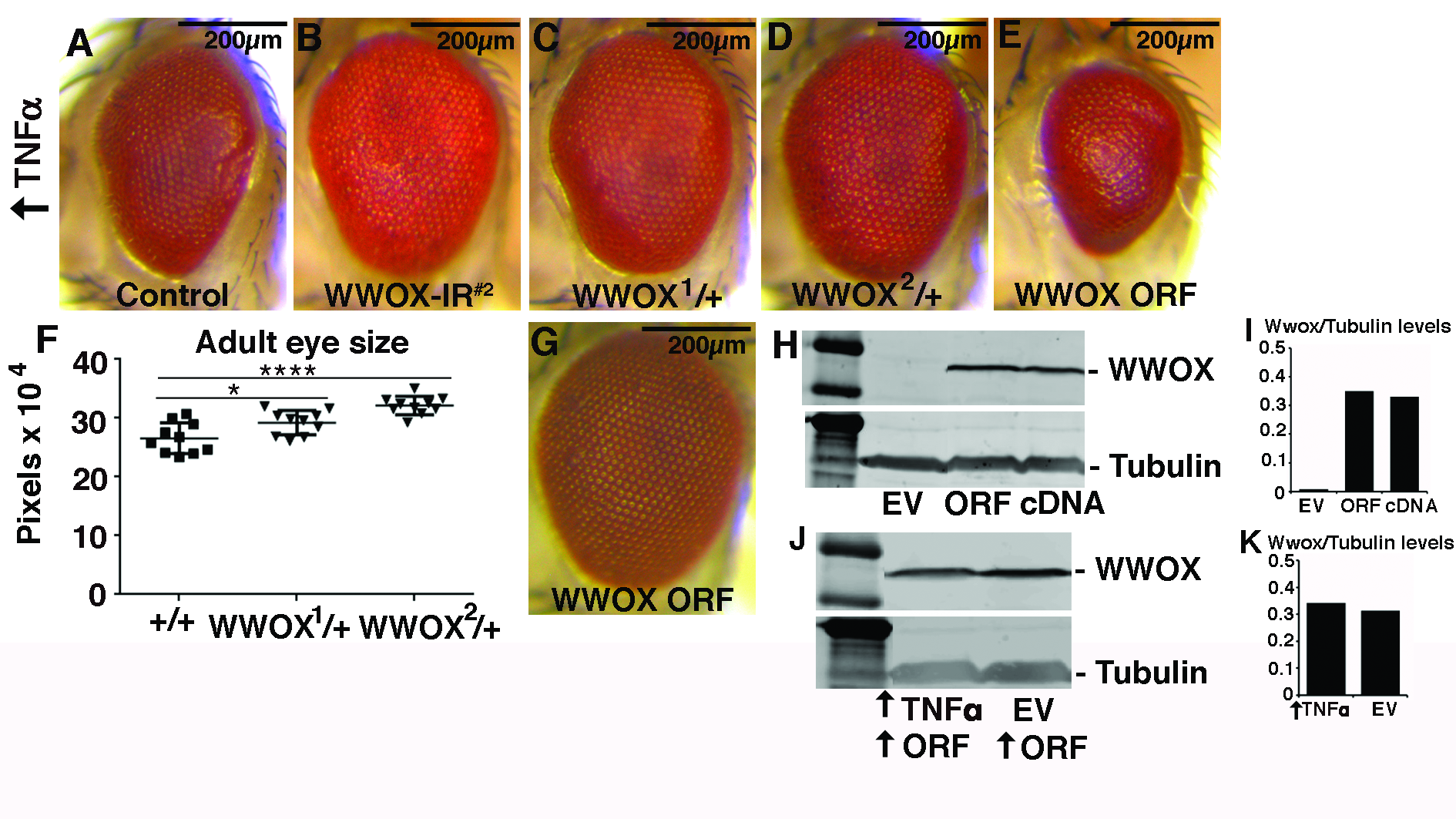

Supplement: S1 Fig — (A) Ectopic expression of Egr/TNFα (GMR>egr +w >+) results in a decrease in eye size and disruption to ommatidial patterning. (B) Decreased expression of WWOX by RNAi knockdown (GMR>egr +w >WWOX-IR #2) resulted in suppression of the rough eye phenotype. (C) Decreased expression of WWOX by heterozygous null allele (GMR>egr +w >WWOX 1 /+) resulted in suppression of the rough eye phenotype. (D) Decreased expression of WWOX by heterozygous insertion mutation allele (GMR>egr +w >WWOX 2 /+) resulted in suppression of the rough eye phenotype. (E) Increased expression of WWOX (GMR>egr +w >WWOX-ORF) resulted an enhancement of the Egr/TNFα phenotype. (F) Quantification of increased eye size with independent heterozygous WWOX alleles (GMR>egr +w >WWOX 1 /+ and GMR>egr +w >WWOX 2 /+). (G) Increased expression of WWOX alone by ectopic expression of the ORF for WWOX (GMR>WWOX-ORF) resulted in no effect on development of the adult eye. (H) Western blot analysis and (I) quantification of the relative levels of WWOX protein expressed in each of the ectopic expression lines compared to a β-Tubulin control. (J) Western blot analysis and (K) quantification of WWOX protein expressed alone and together with Egr/TNFα compared to a β-Tubulin control. (TIF) [file pone.0136356.s001.tif]

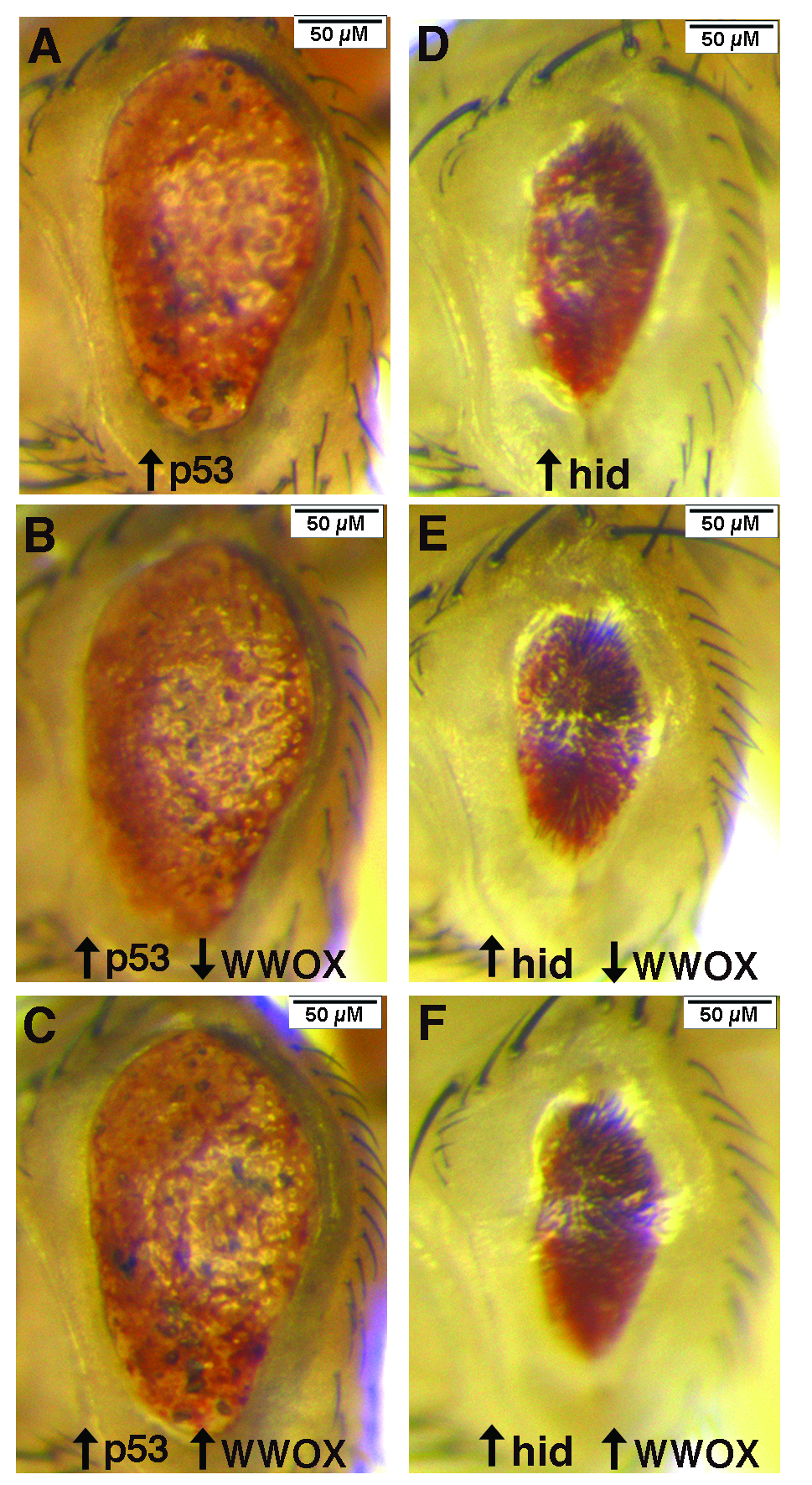

Supplement: S2 Fig — (A) Ectopic expression of Dmp53 in the developing eye (GMR>DmP53>EV) at 18°C in the adult eye results in a phenotype characterized by decrease in eye size and significant disruption to ommatidial patterning accompanied by loss of pigment and the presence of small necrotic lesions. (B) Decreased expression of WWOX by RNAi (GMR>DmP53>WWOX-IR #1 ) resulted in no significant modification. (C) Increased expression of WWOX (GMR>DmP53>WWOX-cDNA) also resulted in no significant modification. (D) Ectopic expression of head involution defective in the adult eye (GMR>GMR-Hid>EV) results in a very strong rough eye phenotype with reduction in eye size and almost complete loss of ommatidial structures. (E) Decreased expression of WWOX by RNAi (GMR>GMR-Hid>WWOX-IR #1 ) resulted in no significant modification. (F) Increased expression of WWOX (GMR>GMR-Hid>WWOX-cDNA) also resulted in no significant modification. (TIF) [file pone.0136356.s002.tif]

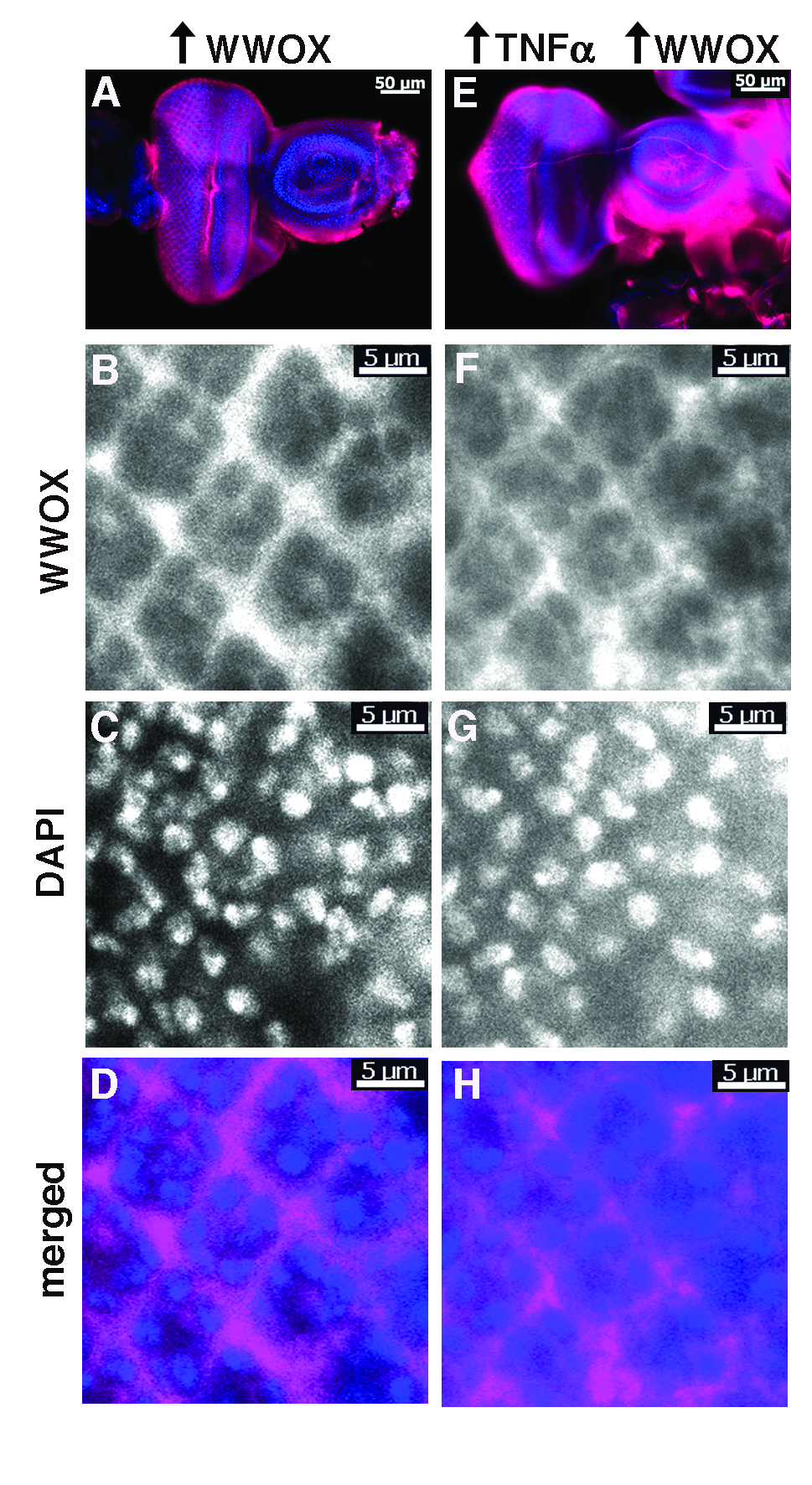

Supplement: S3 Fig — (A-D) Ectopic expression of WWOX alone with GMR-gal4 results in WWOX localisation to areas complementary to the DAPI stained nuclei of eye-imaginal discs of wandering third instar larvae. (E-H) Ectopic expression of WWOX with GMR-gal4 in the presence of ectopic Egr/TNFα expression also results in WWOX localisation to areas complementary to the DAPI stained nuclei of eye-imaginal discs of wandering third instar larvae. (TIF) [file pone.0136356.s003.tif]

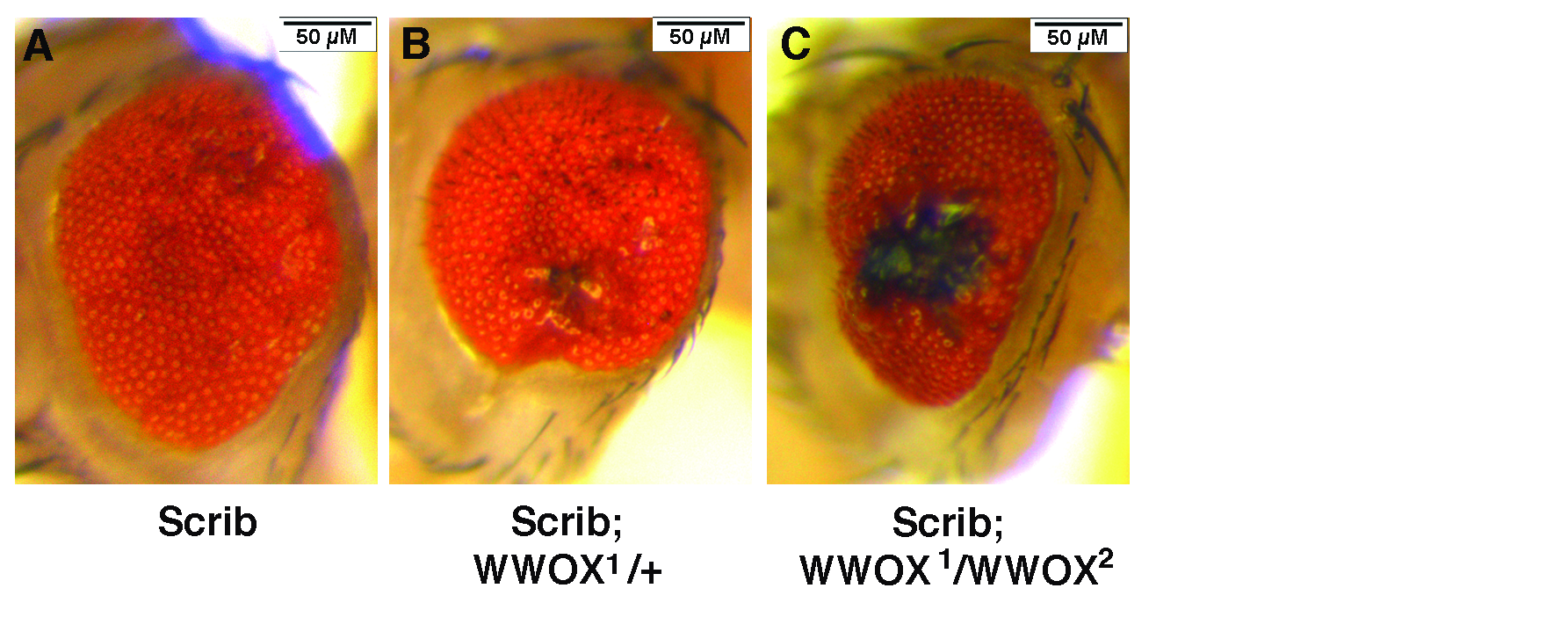

Supplement: S4 Fig — (A) Clones of cells mutant for Scrib generated in the eye using the MARCM system result in a mild adult rough eye phenotype. (B) Decreased WWOX expression throughout the whole animal (Scrib 1; WWOX 1 /+) resulted in a stronger phenotype with a decreased eye size, significant disruption to ommatidial patterning and the presence of some necrotic lesions. (C) Complete absence of WWOX throughout the whole animal (Scrib 1; WWOX 1 / WWOX 2 ) resulted in a phenotype with a decreased eye size, significant disruption to ommatidial patterning and the presence of large necrotic lesions. Genotypes used: Scrib 1 (ey-FLP1, UAS-mCD8-GFP; +/+;tub-GAL4 FRT82B tub-GAL80/ FRT82B scrib 1 ), Scrib 1;WWOX -/+ (ey-FLP1, UAS-mCD8-GFP; WWOX 1 /+;tub-GAL4 FRT82B tub-GAL80/ FRT82B scrib 1 ), Scrib 1; WWOX -/- = (ey-FLP1, UAS-mCD8-GFP; WWOX 1 / WWOX 2;tub-GAL4 FRT82B tub-GAL80/ FRT82B scrib 1). Adults carrying WWOX mutations were generated by crossing ey-FLP1, UAS-mCD8-GFP;;tub-GAL4 FRT82B tub-GAL80/TM6B flies carrying either a WWOX mutant allele or wild-type second chromosome together with FRT82B, Scrib 1 carrying a WWOX mutant allele. (TIF) [file pone.0136356.s004.tif]
